# Peptide-Based Fluorescent Biosensing System for the Detection of the Melanoma Biomarker S100B

**DOI:** 10.1021/acs.bioconjchem.5c00337

**Published:** 2025-11-06

**Authors:** Eleni Chatzilakou, Yubing Hu, Othman Al Musaimi, Lucia Lombardi, Oscar M. Mercado-Valenzo, Nan Jiang, Daryl R. Williams, Ali K. Yetisen

**Affiliations:** † Department of Chemical Engineering, 4615 Imperial College London, South Kensington, London SW7 2BU, U.K.; ‡ Centre for AIE Research, Guangdong Provincial Key Laboratory of New Energy Materials Service Safety, College of Materials Science and Engineering, Shenzhen University, Shenzhen 518060, China; § Faculty of Medical Sciences—School of Pharmacy, 12186 Newcastle University, Newcastle upon Tyne NE17RU, U.K.; ∥ Orthogonal Peptides Limited, London SW7 2AZ, U.K.; ⊥ School of Biological Sciences, Queen’s University Belfast, 19 Chlorine Gardens, Belfast BT9 5DL, U.K.; # West China School of Basic Medical Sciences & Forensic Medicine, Sichuan University, Chengdu 610041, China

## Abstract

Cutaneous melanoma, responsible for 80% of skin cancer mortality, presents urgent diagnostic challenges due to insufficient early detection methods. Current clinical methods rely on invasive biopsies, while noninvasive approaches primarily serve as adjunctive decision-support tools rather than definitive diagnostics. Here, a peptide-based fluorescent biosensing system was developed for the sensitive and rapid detection of S100B, a key prognostic biomarker for melanoma. Our system employs a fluorescently labeled peptide beacon designed for Förster resonance energy transfer (FRET)-based detection, achieving a subnanomolar detection limit (∼0.045 nM) and great selectivity in human serum samples. Peptide synthesis was performed using optimized solid-phase protocols, enabling precise sequence assembly, while the peptide sensor offers efficient detection, lower costs, and high specificity through tailored peptide–protein interactions. The biosensing probe employs complementary peptide nucleic acid (PNA) interactions to achieve proximity-induced fluorescence quenching in the absence of S100B, which reverses via structural rearrangement upon specific S100B binding for accurate quantification. Computational and experimental optimization of the synthetic process has enhanced binding efficiency, sensitivity, and response time–crucial parameters for melanoma-specific detection. By integrating advanced molecular design with optical biosensing, this mechanism aims to enhance the accuracy and accessibility of melanoma diagnostics, ultimately addressing healthcare disparities and improving patient outcomes.

## Graphical abstract

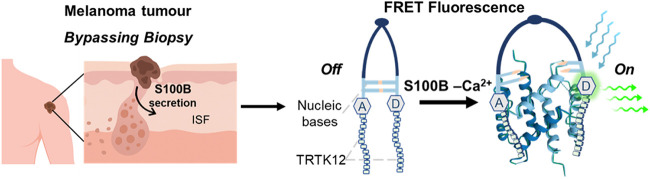

## 1 Introduction

Skin cancer is among the most prevalent malignancies globally, with incidence rates rising significantly since the late 20th century.^1^ In 2020, over 1.5 million cases were diagnosed worldwide, and this number is projected to exceed 2.3 million by 2040.^2^ Cutaneous melanoma is the most lethal form, accounting for 80% of skin cancer deaths due to its high metastatic potential.^3^ While emerging diagnostic tools surpass traditional morphological evaluation in accuracy, they are primarily clinical decision support systems, as skin biopsy remains the gold standard for definitive diagnosis before treatment.^4^ However, biopsies are costly, labor-intensive, and associated with patient discomfort, infection risks, and limited suitability.^5^ Fluorescent biosensors are emerging as transformative point-of-care tools, offering rapid and accurate diagnostics with exceptional sensitivity, selectivity, and portability.^6^ Among these, peptide-based systems stand out as highly effective bioreceptors, particularly for protein detection.^7^ Peptides mimic natural binding motifs, enabling precise, real-time monitoring of biomarkers with minimal interference in complex biological environments.^8^ Their unique biochemical properties–high specificity, biocompatibility, and tunability– extend beyond diagnostics to therapeutic applications. Since 2015, the U.S. Food and Drug Administration (FDA) has approved 36 peptide-based drugs for diverse medical applications, including cancer treatment, cancer imaging, diabetes management, and obesity control.^9–12^ This growing clinical adoption highlights the versatility of peptides as both sensing elements and drug delivery agents; however, challenges remain in the precise synthesis of peptides, particularly in developing efficient methods for incorporating fluorophores and other modifications.^10,13^ Addressing these limitations is essential for enhancing peptide stability and specificity, thereby advancing their broader application in diagnostics and therapeutics.

S100B is a key melanoma biomarker with significant utility in diagnosis, prognosis, and monitoring therapeutic efficacy.^14–16^ Elevated serum S100B levels are strongly associated with metastasis risk, while advanced-stage patients exhibit notably higher levels (e.g., 0.2833–10.52 nM) compared to early stages (e.g., 0.0219–0.1524 nM).^15,17,18^ Concurrently, S100B is a versatile biomarker associated with a range of diseases beyond melanoma, playing a significant role in neurodegenerative disorders (NDDs), traumatic brain injury (TBI), neural and inflammatory conditions, highlighting its broad clinical relevance.^19–21^ Its low concentrations in interstitial fluid and serum pose a challenge for detection despite its critical role in predicting melanoma progression and treatment response.^14,17,22^ Current methods for S100B detection in melanoma primarily rely on antigen–antibody interactions and peptide-based recognition strategies, leveraging a variety of biosensing platforms. For instance, microneedles functionalized with antihuman S100B antibodies have been used in combination with a blotting technique to qualitatively define S100B levels.^23^ Another strategy involves an electrochemical immunosensor based on a chitosan/reduced graphene oxide (CS–rGO) nanocomposite, which offers improved sensitivity and stability through the integration of nanomaterials.^24^

Peptide-based approaches have also shown promise. An electrochemical assay leveraging a 1:2 binding ratio between S100B and a peptide designed for specific recognition uses a capture peptide for biorecognition and a Gly-His-Lys-modified signal peptide for amplification, enhancing detection accuracy in human blood samples.^25^ It highlights the potential of peptide biosensing platforms to achieve sensitivity, selectivity, and stability. However, challenges persist in enhancing the reliability and efficiency of these methods for clinical applications, particularly in the seamless integration of peptide biorecognition units with fluorescent sensing units, especially the bifunctional carboxyfluorescein, which remains a significant hurdle.^26^

Amid these advancements, TRTK12 emerges as a highly promising biorecognition element for S100B detection.^27–29^ With its exceptional binding affinity (*K*_d_ ∼ 260 nM) and specificity for S100B, TRTK12 surpasses all existing peptides in performance.^30^ It interacts with S100B through a combination of hydrophobic and electrostatic interactions, stabilizing the protein structure and enhancing calcium-ion-binding affinity.^29^ Unlike other peptides, TRTK12 shows minimal cross-reactivity with other S100 proteins, further underscoring its potential.^31^ By integrating TRTK12 into advanced optical biosensing systems, future diagnostic tools could achieve high sensitivity, specificity, and clinical utility in detecting and monitoring S100B levels in melanoma.

Herein, we developed a highly sensitive detection platform for the melanoma biomarker S100B, where a fluorescently labeled peptide-nucleic acid (PNA) beacon, with the peptide TRTK12 as the biorecognition element, has been engineered. To overcome synthetic challenges and facilitate fluorophore-modified peptide beacons, we developed an efficient route for incorporating carboxyfluorescein into peptide-based detection systems. The system exploits the dimeric structure of S100B by incorporating a PNA beacon with dual fluorescently labeled peptide arms, each designed to bind one subunit of the S100B homodimer. FRET between the selected fluorophores facilitates an ultrasensitive limit of detection. The detection mechanism relies on intramolecular interactions between complementary PNA bases, bringing the peptide arms into spatial proximity and quenching donor fluorescence upon target binding.

## 2 Results and Discussion

### 2.1 Bioreceptor Design and Molecular Modeling

To develop a fluorescent bioreceptor for the clinically significant biomarker S100B in cutaneous melanoma (Figure 1A), a peptide beacon was designed employing 5-carboxyfluorescein (5-FAM) paired with Dabcyl, a cost-effective quencher that yields robust FRET signals. For the synthetic route, the commonly used maleimide-cysteine thiol-Michael coupling, which suffers from low yields due to side reactions with the Pbf protecting group on arginine, was replaced with a Cu-catalyzed click reaction (CuAAC) to reliably join the two peptide arms at larger scales.^32,33^ Furthermore, the incorporation of glycine residues at the termini of each arm enhances structural flexibility, optimizing the spatial configuration for binding the dimeric S100B protein. Under high Ca^2+^ conditions, S100B undergoes a conformational change, transitioning from the apo-S100B to the Ca^2+^-S100B form, leading to exposure of binding sites that can be identified by the TRTK12 sequence, enabling interactions with the peptide nucleic acid (PNA) beacon as shown in Figure 1B-i.^29,34^ The complementary base pairing of the PNA bases that were integrated into the peptide sequence keeps the two arms of the fluorescent peptide beacon in spatial proximity, with one arm carrying a donor fluorophore (5-FAM) and the other an acceptor (Dabcyl) (Figure 1B-ii). This configuration quenches fluorescence emission from the donor due to the proximity of the acceptor. Upon binding to the symmetrically related binding sites of S100B, FRET occurs, resulting in increased fluorescence from the donor, detectable via a spectrophotometer. The FAM-Dabcyl FRET system offers strong fluorescence from FAM and efficient quenching by Dabcyl, a dark quencher, ensuring minimal background interference while being significantly cost-effective compared to other FRET systems.^35^ The FRET efficiency, dependent on the Förster radius and the donor–acceptor distance, is achieved by hydrogen bonds (≈0.2 nm) between the PNA bases, ensuring a suitable FRET range.^36^ Each arm of the fluorescently labeled PNA beacon carries a peptide chain, namely TRTK-12, that binds to one of the subunits of the homodimeric S100B protein and functional moieties, as shown in Figure 1B-iii.

**Figure 1:**
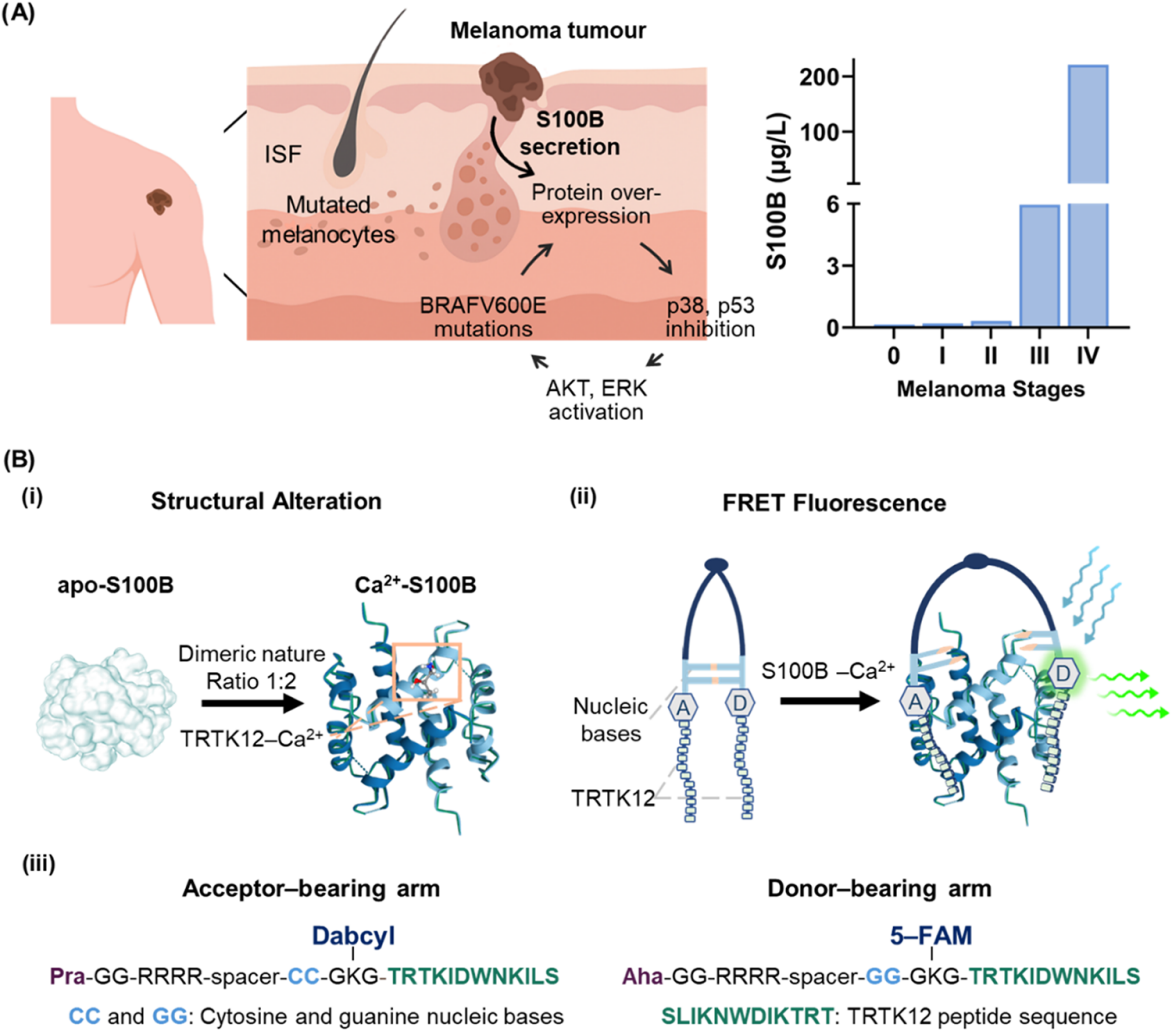
Mechanistic role of S100B in melanoma progression and its detection via a custom PNA-based fluorescent biosensing system (A) Schematic illustration of the mechanistic role of S100B in tumor biology and its marked upregulation across disease stages. (B) Biosensing mechanism of the PNA fluorescent beacon illustrating the (i) calcium-induced structural changes in S100B expose TRTK-12-specific binding sites, (ii) complementary base-pairing of the fluorescent peptide beacon quenches fluorescence, while FRET activation upon binding to S100B increases donor fluorescence, (iii) detailed residue composition of the beacon arms.

#### 2.1.1 Design and Computational Simulation of Peptide Sequence

Figure 2A illustrates the rationale behind our molecular design. Each component was strategically selected to ensure structural integrity, biocompatibility, and efficient functionalization. Complementary PNA bases (G–C) enable stable intramolecular hybridization,^37^ while glycine spacers and O-linkers confer flexibility and reduce steric hindrance,^38^ facilitating efficient bioconjugation. The incorporation of L-azidohomoalanine ensures compatibility with hydrazine treatment,^39,40^ preserving downstream click reactivity (see Sections 3.2 and 3.5). Linking the arms at the N-terminal ensured the protein identification peptide sequence remained accessible, preventing sequestration within a closed loop.

**Figure 2:**
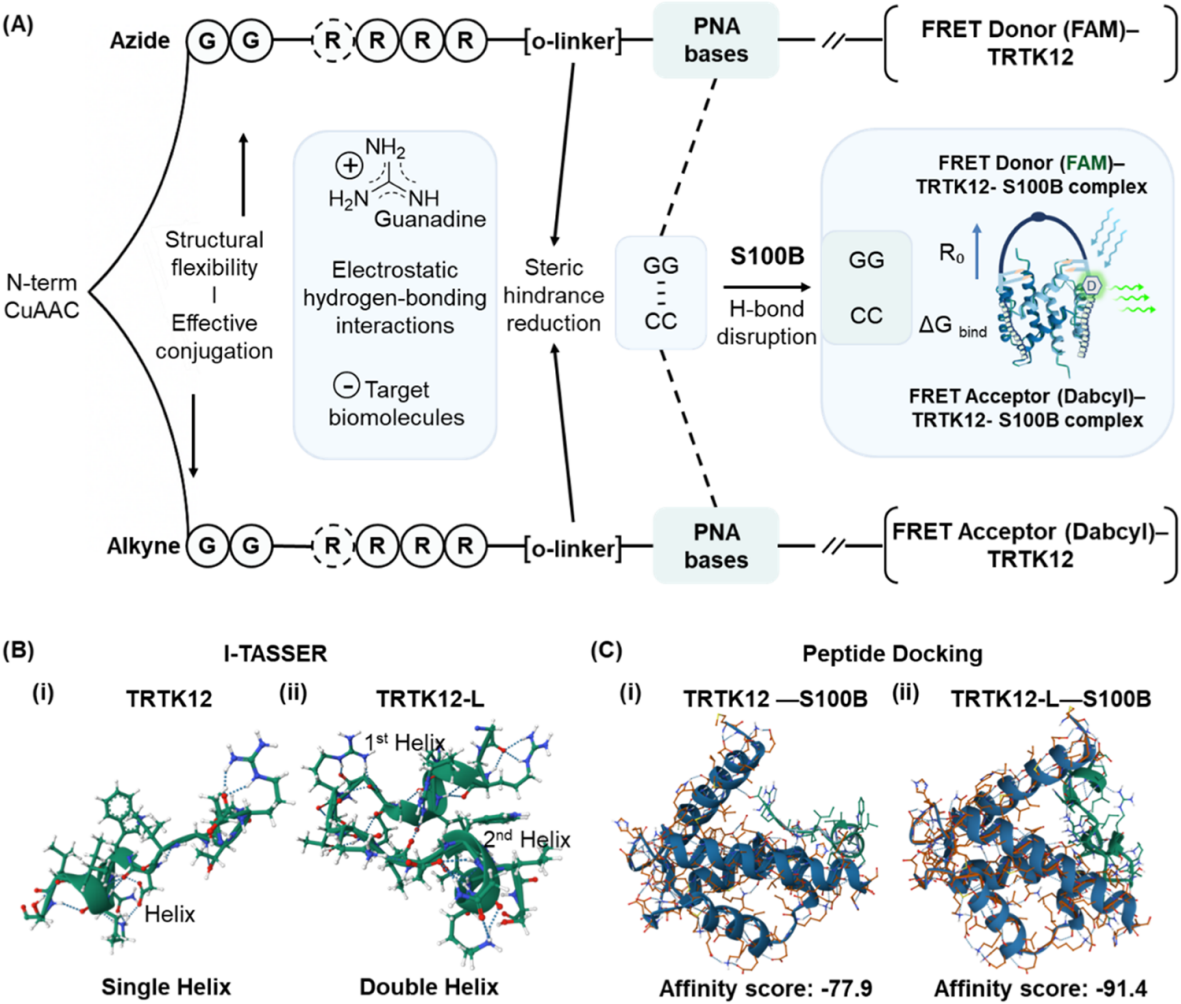
Bioreceptor design and molecular modeling results. (A) Molecular design strategy highlighting the key functionalities of the deployed chemical residues. (B) Structural predictions and helicity illustrations of TRTK12 (i) and TRTK12-L (ii) generated by I-TASSER. (C) Docking results from HADDOCK, highlighting the top-ranked clusters and the most likely interaction models between S100B and the respective peptides.

Before proceeding with the synthesis, the theoretical foundation of our design through molecular modeling was validated to assess its structural and functional advantages over TRTK12. Using I-TASSER for protein structure prediction and MolProbity for stereochemical structural validation, TRTK12-L was analyzed, given I-TASSER’s limitation to natural amino acids. Results confirmed that TRTK12-L adopts a more pronounced α-helical conformation, enhancing stability and specificity in bioreceptor interactions (Figures 2B and S1). Helical structures improve recognition efficiency through multivalent interactions and amplify detection signals via conformational changes.^41,42^

Ramachandran analysis further confirmed TRTK12-L’s structural integrity. Compared to TRTK12, its extended sequence introduces a stable loop region (residues 1–13) and reinforces α-helical stability (residues 14–20), as indicated by improved dihedral angles and Z-scores (Tables S1 and S2). These enhancements contribute to a structurally optimized peptide with superior stability and flexibility.

To evaluate TRTK12-L’s binding potential to S100B, HADDOCK docking analysis was performed, incorporating calcium-dependent interaction restraints. TRTK12-L exhibited consistently lower HADDOCK scores (e.g., −91.4 ± 4.3 vs −77.9 ± 2.3 for TRTK12), stronger electrostatic interactions, and a larger buried surface area, indicating enhanced affinity and stability (Figure 2C and Tables S3 and S4). These findings confirm TRTK12-L’s superior interaction properties, supporting its potential as a high-potential bioreceptor for S100B detection in melanoma diagnostics. Encouraged by these results, peptide synthesis and experimental validation were completed.

### 2.2 Synthesis and Optimisation of the Bioreceptor

During the synthesis of the TRTK12 starting sequence, a custom-built coupling difficulty prediction tool was used to guide the coupling of leucine and tryptophan, which are part of the sequence, requiring 1 h per coupling with double coupling due to their predicted difficulty. For subsequent steps, a stepwise approach was implemented to determine the optimal reaction times for each component, focusing on maximizing yield, minimizing coupling inefficiencies, and reducing overall costs. Ninhydrin testing and sample-based cleavages, followed by HPLC and MS analysis, were conducted at each step to confirm complete coupling and verify the correct synthetic product. Coupling reactions were performed under the optimized conditions outlined in Table 1.

**Table 1:** Optimized Coupling Conditions for PNA-Beacon Synthesis

chemical moiety	coupling reagent	reaction time (h)	coupling strategy
tryptophan	DIC/oxymapure	1	double coupling
leucine	DIC/oxymapure	1	double coupling
lysine (Dabcyl)	DIC/oxymapure	1	double coupling
lysine (IvDde)	DIC/oxymapure	1	double coupling
glycine	DIC/oxymapure	1	double coupling
PNA Monomers	DIC/oxymapure	2	single coupling
NH-PEG2-CH_2_COOH	DIC/oxymapure	1	quadruple coupling
arginine	DIC/oxymapure	1	double coupling
Fmoc-Pra–OH	PyBOP/DIPEA	1.5	double coupling
Fmoc-L-Aha	PyBOP/DIPEA	1.5	double coupling

On the dabcyl-bearing arm, lysine residues were introduced with Dabcyl-*N*-succinimidyl ester (1 h per coupling, double coupling), followed by the addition of glycine, PNA­(C) monomers, 8-amino-3,6-dioxaoctanoic acid (NH-PEG2-CH_2_COOH), four arginines (1 h each, double coupling), two additional glycines (1 h each, double coupling), and Fmoc-Pra–OH (1.5 h each, double coupling). In the case of the 5-FAM-bearing arm, similar steps were followed, with lysine protected by 4-(4,4′-dimethoxytrityl) oxycarbonyl (IvDde) and the same coupling strategy employed for the PNA­(G) monomers and the other moieties. The coupling of propargylglycine (l-Pra) and azidohomoalanine (l-Aha) was carried out using benzotriazol-1-yloxytripyrrolidinophosphonium hexafluorophosphate (PyBOP) (3 equiv) and *N*,*N*-diisopropylethylamine (DIPEA) (6 equiv), avoiding side reactions associated with harsher reagents like DIC and preserving the integrity of these functional groups. Following this, the Fmoc protecting group of l-Aha was removed and replaced with a Boc group using di*tert*-butyl decarbonate and DIPEA in dichloromethane (DCM). The PNA monomers were prepared in peptide-grade *N*-methylpyrrolidone anhydrous (NMP) (0.2M) to ensure complete dissolution and were preactivated in situ for 3 min before their addition to the main reaction mixture.

#### 2.2.1 Optimisation of IvDde Removal Protocol

Optimising hydrazine treatment for IvDde removal is crucial to ensure complete removal while minimizing lysine ornithination and preserving peptide integrity and biological function. Initial deprotection trials employing 2% hydrazine in 12.5 mL per gram of resin (single treatment for 10 min) achieved partial IvDde removal, with approximately 50% deprotection efficiency (Figure S2-i). To enhance reaction efficacy, the volume of 2% hydrazine was increased to 75 mL per gram of peptide resin to improve surface area exposure of the resin beads. However, even with three consecutive 3 min treatments (3× for 3 min each), IvDde removal remained incomplete (Figure S2-ii). To achieve complete removal, the hydrazine concentration was increased to 4% in 75 mL per gram of peptide-resin. Under these conditions, full IvDde removal was accomplished using two 3 min treatments followed by one 5 min treatment (2× for 3 min, 1× for 5 min). However, the higher hydrazine concentration correlated with increased lysine or arginine ornithination, as determined by mass spectrometry. Ornithination was quantified at 21.47% under these optimized conditions, compared to 9.7% and 16% for 2% hydrazine treatments at 12.5 mL (1× for 10 min) and 75 mL (3× for 3 min), respectively. To minimize ornithination, low-temperature (8 °C) deprotection conditions were explored with 4% hydrazine. Single and triple treatments (1× and 3× in 75 mL per gram of peptide-resin) were tested. While a reduction in reaction temperature led to marginal decreases in ornithination (8.47% for 1× and 19.03% for 3× treatments), these conditions failed to achieve complete IvDde removal (Figures S2-iii, S3 and S4). Ultimately, complete deprotection was best achieved with 4% hydrazine at room temperature using two 3 min treatments followed by one 5 min treatment (2× for 3 min, 1× for 5 min). This protocol resulted in a trade-off of increased ornithination (∼20%) but provided an optimized balance between efficient IvDde removal and minimal side reactions. The optimized protocol enables reliable Aha-FAM peptide functionalization with minimal impact on integrity, improving purity, yield, and suitability for sensing applications, given the importance of lysine and arginine residues in the structure.

#### 2.2.2 Optimisation of FAM Coupling

The coupling of 5-carboxyfluorescein (5-FAM) to peptide chains in solid-phase peptide synthesis (SPPS) posed significant challenges due to the dye’s bifunctionality, leading to esterification and the formation of doubly labeled products during initial experiments using standard coupling conditions (3:3:3 molar ratio of FAM:DIC: OxymaPure) (Figure S5). Lower reagent ratios (e.g., 0.5:4:2 and 2:4.5:4.5) failed to achieve sufficient coupling efficiency, producing incomplete reactions and persistent side products (Figure S6).

Computational simulations using DMol^3^ in Materials Studio (Figure S7) to examine the reagent addition order effect based on reaction kinematics as shown in Table 2, revealed that coupling FAM before azide addition (Scenario 1) resulted in three energetically favorable byproducts: doubly labeled peptides with azide either on the *N*-terminal or side chain, and singly labeled peptides with azide on the side chain. These byproducts exhibited lower activation energies (*E*_A_) and reaction energies (*E*) compared to the desired product (*E*_A_ = 242.97 kcal/mol; Δ*E* = 38.34 kcal/mol), increasing their likelihood of formation. Conversely, coupling FAM after azide addition (Scenario 2) produced only one byproduct, a doubly labeled peptide, which had a higher activation energy and was less kinetically favored.

**Table 2:** Energetic Analysis of Reaction Pathways for FAM Integration Scenarios Performed in Materials Studio

product	*E*_A_ (kcal/mol)	Δ*E* (kcal/mol)
1st Scenario: Azide Addition before Dye Introduction		
main (desired product)	242.97	38.34
byproduct A (doubly labeled, N-terminal azide)	417.86	26.49
byproduct B (doubly labeled, side chain azide)	160.32	60.52
byproduct C (singly labeled, side chain azide)	189.11	38.93
2nd Scenario: Dye Addition before Azide Introduction		
main (desired product)	242.97	38.34
byproduct A (doubly labeled, N-terminal azide)	417.86	26.49

Experimentally, the azide-first strategy with optimized conditions (1:1:1 molar ratio of FAM:DIC:OxymaPure under acidic conditions for 2 h) yielded >60% of the desired product, significantly reducing byproduct formation. Alternative strategies, such as coupling FAM to lysine side chains using Mtt or using PyBOP/DIPEA, exhibited limitations due to dye aggregation under basic conditions. The final synthesis under the azide-first approach involved sequential coupling, starting with azide integration, Boc substitution, and hydrazine treatment, followed by FAM addition, which minimizes esterification and ensures efficient dye incorporation. This approach was validated through HPLC/MS analysis, which confirmed a predominant peak corresponding to the singly labeled product, as presented in Figures S8 and S9.

### 2.3 Purification

The linear gradient elution method proved to be superior for scaled-up purification, yielding consistent separation and high-purity products despite some loss of yield. This method was successfully applied to the purification of both individual arms and the beacon. Analysis of the collected fractions identified a prominent peak corresponding to the beacon, along with peaks associated with known impurities. The primary impurities included unlabeled products and doubly labeled species, particularly in the case of the FAM arm, where partial ornithination was determined. For the semipreparative purification of the beacon, excellent separation and high purity were achieved, with the resulting material being freeze-dried for subsequent sensing applications. Here, purity was prioritised over yield during purification, recognizing the stringent purity requirements for subsequent bioconjugation. This approach minimized downstream complications, ensuring compatibility with the biomedical nature of the application. For both arms, the achieved purity was ≥99% (Figures 3 and S10).

**Figure 3:**
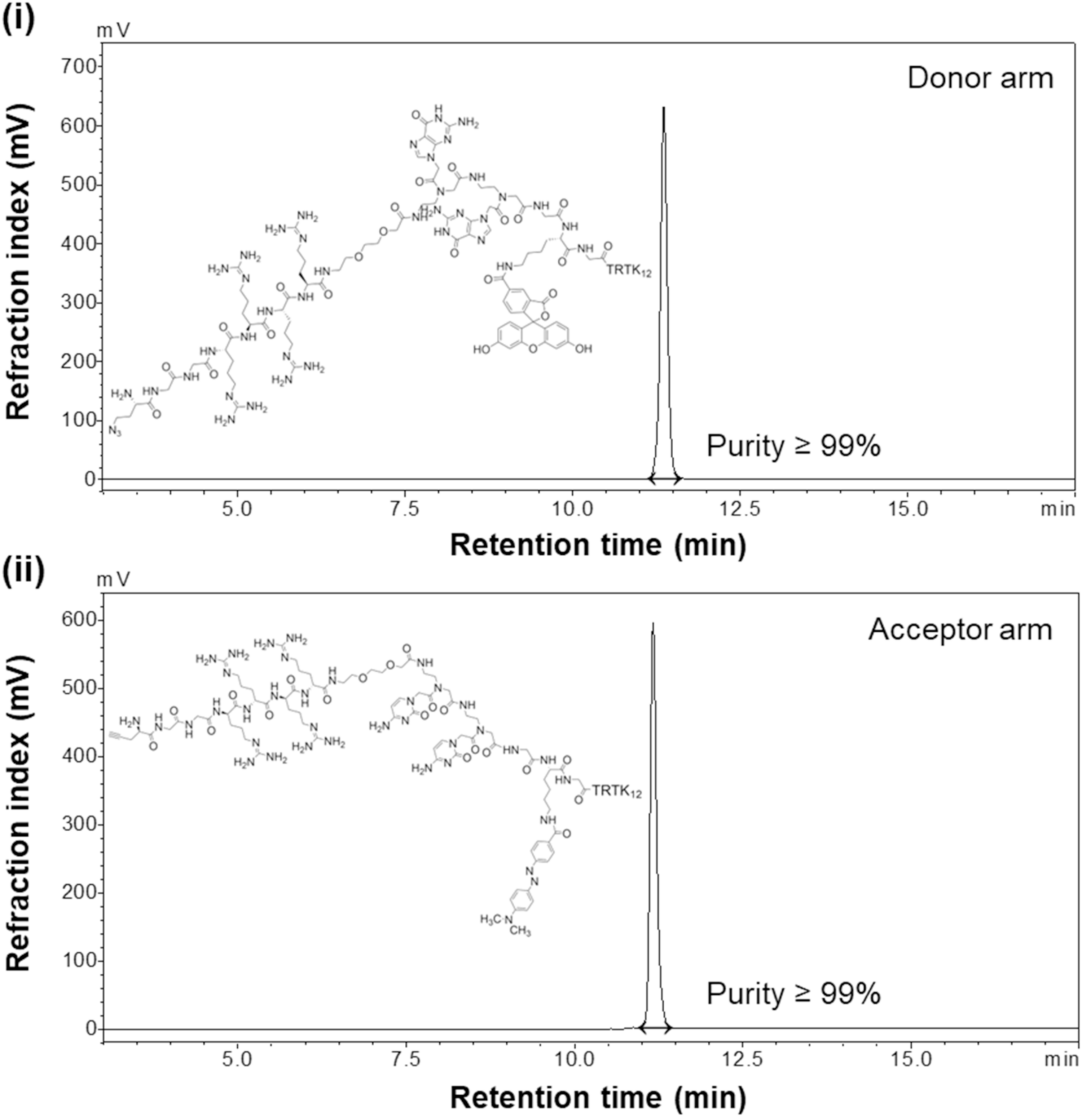
HPLC chromatograms of the purified peptide arms after the completion of individual syntheses are shown, showcasing (i) the 5-FAM-bearing arm and (ii) the Dabcyl-bearing arm, highlighting ≥99% purity.

### 2.4 CuAAC Bioconjugation

CuAAC orthogonal reaction, ideal for peptide conjugation, operates in mild aqueous conditions to produce stable, hydrolysis- and enzymatic-resistant 1,4-disubstituted triazoles. Compared to the thiol-Michael addition, it offers superior specificity, scalability, and biomolecular compatibility.^43^ The optimized conjugation efficiently coupled the two peptide arms into a unified peptide beacon after 4 h under optimized reaction conditions (Figure S11), as seen by the HPLC-MS analysis in Figure 4 with the only products corresponding to the beacon and traces of the arm in excess. Although copper­(II) sulfate pentahydrate is not catalytically active on its own, the introduction of sodium ascorbate allows in situ reduction to copper­(I).^43^ This reduction is critical for generating the active catalytic species, thereby enhancing the reaction efficiency and minimizing side reactions. Tris­(3-hydroxypropyltriazolylmethyl) amine (THPTA) was selected over tris ((1-benzyl-4-triazolyl)­methyl) amine (TBTA) for its solubilizing properties in water that promote the formation and stability of the copper acetylide intermediate. The inclusion of aminoguanidine, primarily used to scavenge advanced glycation end-products (AGEs) in biological contexts, was also used in this context to address potential glycation on arginine residues due to the ascorbate.^44,45^ The strict control of reagent volumes, in addition to their concentrations and molar equivalents, was essential to avoid perturbations in ionic strength and pH that could compromise reaction performance. Furthermore, the decision to use an excess of the azide-bearing arm was based on preliminary observations that an excess of the alkyne-bearing arm (dabcyl-modified) tended to form dimers and aggregate, thereby inhibiting the reaction. Overall, the optimized conditions—along with the standard protocol of pH 7.0 buffer and controlled reagent addition under a nitrogen atmosphere at an elevated temperature (45 °C)–ensured rapid reaction kinetics, efficient catalyst solubility, and successful conjugation of the peptide arms.

**Figure 4:**
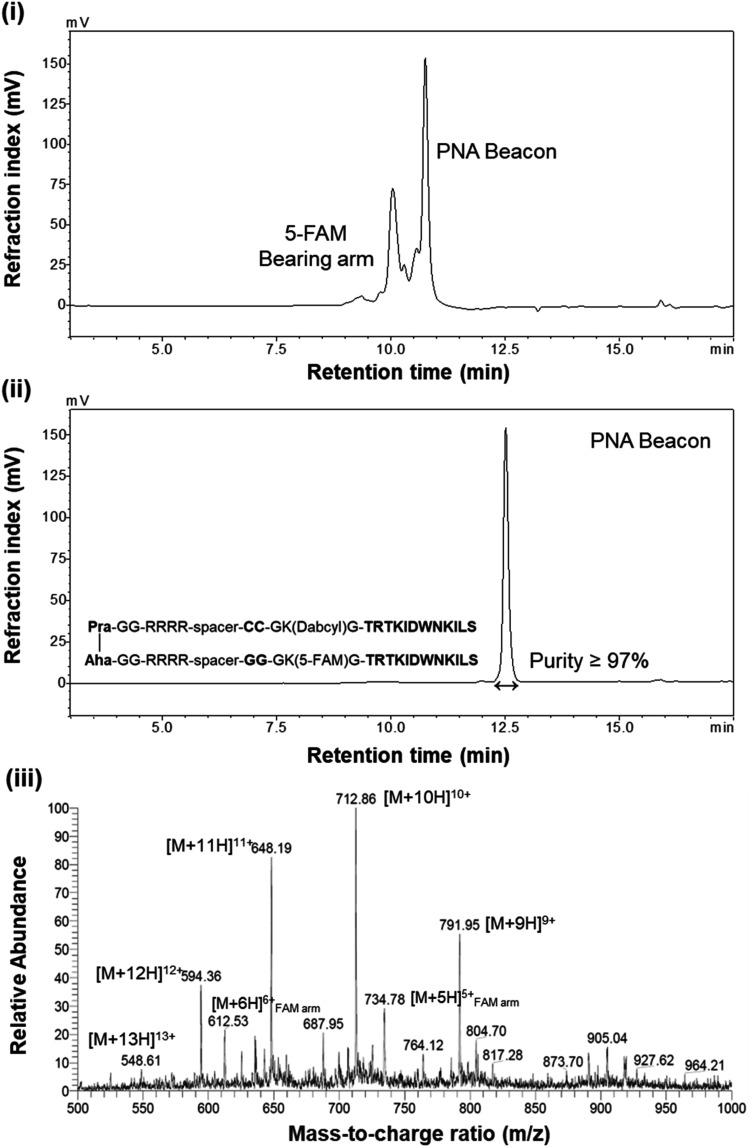
Comprehensive characterization of the bioconjugation reaction products: (i) HPLC chromatogram of the reaction mixture after 4 h under optimal conditions, (ii) HPLC chromatogram of the purified beacon, and (iii) MS analysis confirming successful bioconjugation with exceptionally high purity, showing minimal excess of the 5-FAM-bearing arm fragments.

### 2.5 Peptide-Based Sensing for S100B Detection

To establish the optimal conditions for fluorescence signal acquisition, preliminary experiments were conducted to evaluate the effects of bioreceptor concentration. Fluorescence measurements were recorded at 25, 30, and 35 °C using an initial bioreceptor concentration of 1 μM and S100B protein concentrations of 0, 20, and 80 nM (Figure 5-i). Across all tested conditions during this preliminary testing, the fluorescence intensity ratio of the 0 nM protein sample was higher than that of samples containing protein, except at 35 °C with 80 nM of protein, suggesting significant bioreceptor autofluorescence that masked the fluorescence stemming from the bioreceptor-protein interaction. This observation indicated that a receptor concentration of 1 μM resulted in fluorescence quenching upon protein interaction, likely due to excessive fluorophore proximity and self-quenching effects.

**Figure 5:**
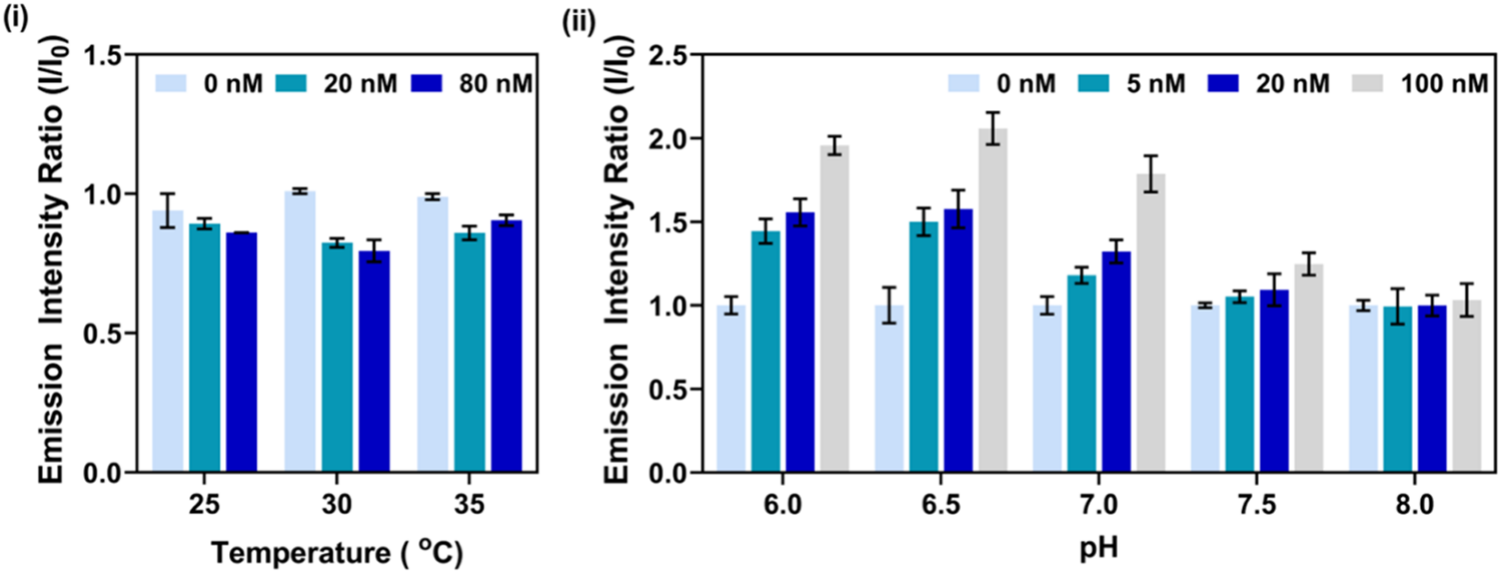
Optimisation of experimental conditions for S100B sensing: (i) Effect of temperature on fluorescence intensity at 25 °C, 30 °C, and 35 °C, demonstrating enhanced signal differentiation at 35 °C for 1 μM of bioreceptor and significant autofluorescence effect. (ii) Fluorescence intensity ratio as a function of pH (6.0–8.0) at 35 °C for varying concentrations of S100B (0, 5, 20, and 100 nM). Error bars represent the standard errors of three different samples.

To mitigate this issue while preserving sufficient signal sensitivity, a reduced bioreceptor concentration of 500 nM was selected for subsequent experiments. This concentration minimized autofluorescence while ensuring robust fluorescence detection within the instrument’s sensitivity range. At 500 nM of bioreceptor, the 0 nM of protein sample consistently exhibited the lowest fluorescence intensity, validating this concentration as the optimal working condition. Furthermore, the strongest fluorescence signals were observed at 35 °C across all protein concentrations, aligning with physiological skin temperature (33.5–36.9 °C), reinforcing its suitability for potential transdermal applications.^46^

The performance of the bioreceptor for S100B detection was found to be highly pH-dependent, reflecting the underlying physicochemical nature of the biomolecular interactions involved (Figure 5-ii). At lower pH values, elevated fluorescence intensities were observed even at low analyte concentrations; however, these conditions also introduced significant signal overlap, potentially compromising analytical resolution. In contrast, higher pH conditions resulted in diminished receptor–analyte interactions, as evidenced by reduced signal intensity. While the fluorophore 5-FAM is known to exhibit enhanced fluorescence emission at pH values ≥7,^47^ the observed signal variations across pH were primarily attributed to pH-induced conformational changes in both the target protein and the bioreceptor peptide. Protonation of key residues at acidic pH may promote structural states that favor complex formation and energy transfer. Given that the tumor microenvironment in melanoma typically exhibits an average pH of ∼6.96,^48^ pH 7.0 was selected as the optimal condition, offering a balance between high signal intensity, minimal overlap, and clinical relevance.

With the optimized bioreceptor concentration (500 nM), temperature (35 °C), and pH (7.0), the binding kinetics of S100B protein were assessed over incubation periods of 30, 60, and 90 min using protein concentrations ranging from 0 to 100 nM (Figure 6A). The objective was to determine the optimal interaction time that maximized signal differentiation, particularly at lower protein concentrations, while preventing signal overlap. The time-dependent fluorescence response likely reflects the dynamic equilibrium between the S100B protein and the peptide-based bioreceptor. After 60 min of incubation, a clear correlation emerged between fluorescence intensity and S100B concentration, with minimal signal overlap, indicating optimal differentiation. However, by 90 min, the fluorescence intensity began to decline, particularly at lower S100B concentrations, accompanied by increased signal overlap. This behavior may result from the reversible nature of hydrogen bonding and van der Waals interactions between the target protein and bioreceptor, as well as the intrinsic half-life of S100B, leading to partial dissociation of the complex. Spectral analysis at 30, 60, and 90 min revealed that higher concentrations of S100B (≥50 nM) could be reliably distinguished as early as 30 min, while lower concentrations required longer incubation times to ensure accurate detection.

**Figure 6:**
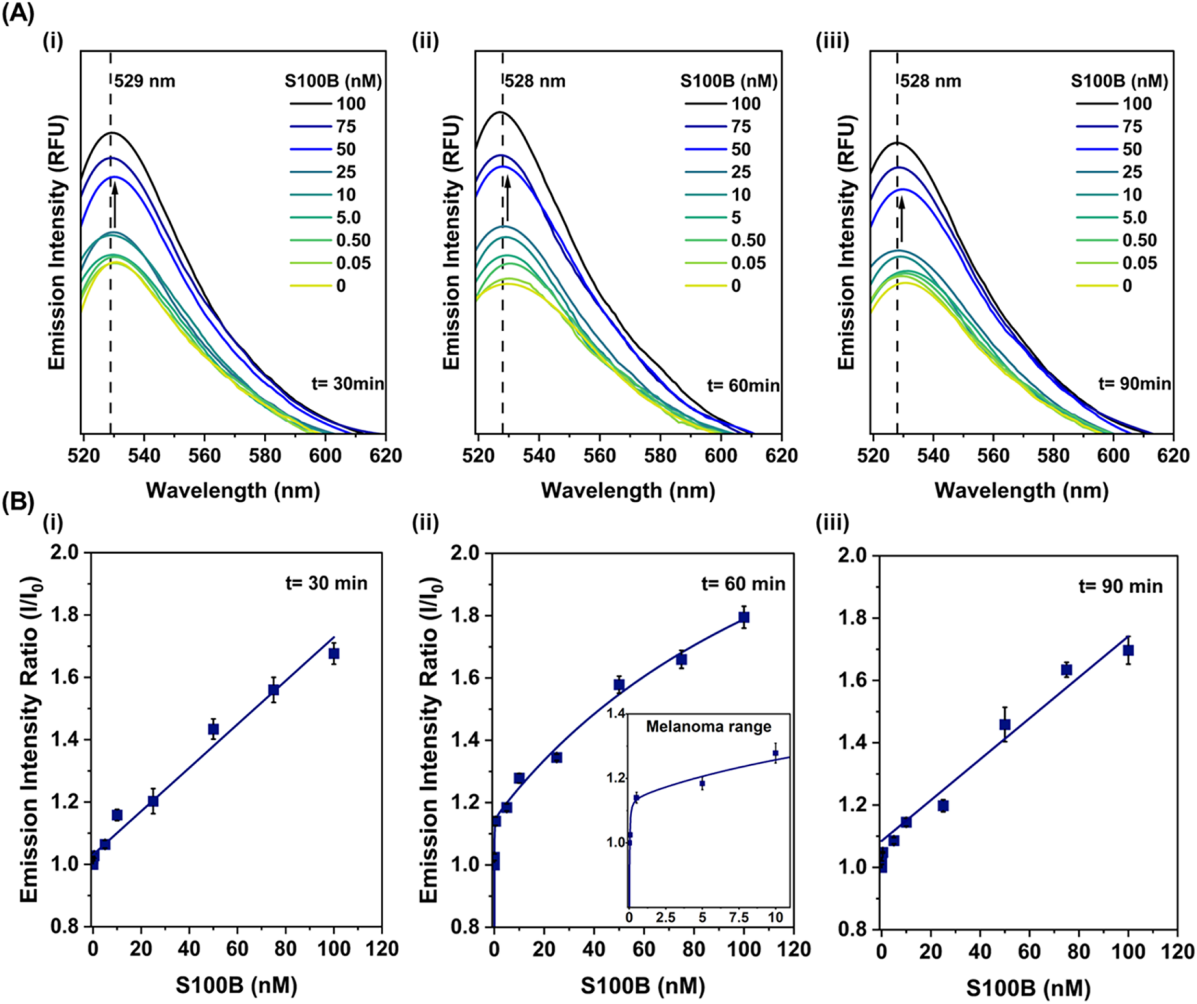
Time-dependent fluorescence response and quantification of S100B detection. (A) Fluorescence emission spectra recorded at (i) 30, (ii) 60, and (iii) 90 min incubation times of the bioreceptor and the protein for varying S100B concentrations (0–100 nM). The emission maximum remained consistent at 528–529 nm across all time points. All spectra were collected by excitation at 499 nm. (B) Calibration curves for fluorescence-based detection of S100B protein at different incubation times and concentration ranges. Data represent mean ± standard error from four independent experiments (*n* = 4).

For each time point, calibration curves were generated to evaluate the relationship between fluorescence intensity and S100B concentration (Figure 6B). At 30 and 90 min, the signal exhibited a linear response (*y* = 1.0471 + 0.01401·[S100B], *R* ^2^ = 0.9628 and *y* = 1.1089 + 0.0129·[S100B], *R* ^2^ = 0.9336, standard curves for the clinically relevant range) across the tested concentration range, likely due to significant signal overlap at <5 nM S100B, limiting the applicability of more complex binding models, as shown in Figure 6B-i,iii. In contrast, for 60 min incubation, a two-site binding model provided the best fit to the data ( $y=0.9945-0.005201\cdot \left[S100B\right]\frac{3.34\cdot \left[S100B\right]}{363.6+\left[S100B\right]}+\frac{0.212\cdot \left[S100B\right]}{0.145+\left[S100B\right]}$ , standard curve for the clinically relevant range) (Figure 6B-ii). The two-site binding model accounted for heterogeneous interactions, with a high-affinity site dominating at low concentrations and a secondary, lower-affinity site at higher concentrations. As S100B is a homodimer and the bioreceptor contains two distinct recognition sequences, cooperative or multisite interactions were expected.

The limit of detection (LOD) was calculated as LOD = *Y*_Blank, normalized_ + 3 × σ_Blank_, where *Y*_Blank, normalized_ is the normalized signal in the absence of the analyte and σ_blank_ is the corresponding standard deviation. Using the calibration curve as obtained for *t* = 60 min, the LOD for S100B detection was determined to be 0.045 ± 0.0149 nM. Importantly, the LOD of 0.045 nM is below the main melanoma diagnostic range (0.1524–10 nM),^15,17,18^ (Figure 6B-ii). To evaluate the accuracy and precision of the developed biosensing system, spike-and-recovery experiments were conducted using human serum. The biosensing system exhibited excellent accuracy, with recoveries ranging from 100.5% to 108.0%, and high precision, as reflected by low standard deviations (SD ≤ 0.18, *n* = 3). These results demonstrate reliable quantification and minimal matrix interference, confirming the suitability of the assay in biologically relevant conditions (Table 3).

**Table 3:** Spike and Recovery Results for S100B Quantification in Human Serum after 60 Min

spiked S100B (nM)	recovered S100B (nM)	% recovery (mean)	SD (*n* = 3)
0.5	0.53	106.0	0.0686
5	5.44	108.0	0.1827
10	10.05	100.5	0.0961

## 3 Experimental Procedures

### 3.1 Bioreceptor Design and Modeling

#### 3.1.1 In Silico Structure Prediction Using I-TASSER

Structural models of the peptide sequences and S100B were generated using I-TASSER v.5.1 (Iterative Threading Assembly Refinement) for homology-based protein structure prediction.^49,50^ The sequences were submitted to the I-TASSER web server, and the program was executed using default parameters.^51^ For each sequence, I-TASSER generated up to five structural models, ranked according to their C-score, evaluating accuracy and quality of each predicted structure (ranging from −5 to 2, with higher values indicating greater confidence). The top-ranked models were selected for further analysis.

#### 3.1.2 Ramachandran Analysis Using MolProbity

Structural validation of the predicted peptide models was performed using MolProbity, an online structure-validation tool. The TRTK12-L (GGRRRRGLGTRTKIDWNKILS) and TRTK12 peptide sequences were uploaded to the server, and the program was executed using default parameters. MolProbity v4.4 assesses structural quality by analyzing backbone geometry, steric clashes, and side-chain rotamer conformations.^52^ The Ramachandran analysis was used to evaluate backbone dihedral angles (ϕ, ψ, and ζ), classifying residues into favored, allowed, or outlier regions based on high-resolution protein structures, allowing for the residues to be categorized by structural type (Loop or Helix).^53^

#### 3.1.3 Molecular Docking Using HADDOCK

Docking simulations were performed to model the interaction between S100B and the peptides TRTK12 and TRTK12-L using the HADDOCK (High Ambiguity Driven Biomolecular Docking) web server and were executed using default settings.^54,55^ The examined structures (S100B and TRTK12/TRTK12-L) were generated using I-TASSER (Section 3.1.1). All structures were prepared by removing water molecules, and the active residues were defined based on known interaction sites for the peptide sequence (e.g., 2:Arg, 3:Thr, 4:Lys, 7:Trp, 9:Lys) and the protein (e.g.,7:Ala, 44:Phe, 46:Glu, 47:Glu, 57:Val, 63:Asn, 80:Met, 88:Phe).^29,30,56^ The passive residues were automatically assigned by HADDOCK based on the input structures.

### 3.2 Synthesis and Optimisation of the Bioreceptor

The TRTK12 peptide and the fluorescently labeled beacon were synthesized using the Fmoc/tBu protection strategy, a standard method in peptide synthesis. This approach involves the use of Fmoc (fluorenyl methoxycarbonyl) and tBu (*tert*-butyl) protective groups to prevent side reactions during peptide assembly. The common for both arms peptide sequence OH-SLIKNWDIKTRTG-NH_2_ was created on a solid support with an amide resin (Fmoc-Rink-Amid-4-methylbenzhydrylamine MBHA, 0.4 mmol/g, 431041–83–7, Iris Biotech). The synthesis of peptides followed a standard methodology employed in our laboratory, utilizing 3 equiv of Fmoc-protected amino acids, 3 equiv of OxymaPure (3849–21–6, Novabiochem), and 3 equiv of *N*,*N*-diisopropyl carbodiimide (DIC) (693–13–0, Sigma-Aldrich) in dimethylformamide (DMF) (68–12–2, Sigma-Aldrich) unless stated otherwise.^57^ All amino acids were purchased from Merck/Sigma-Aldrich (U.K.), Iris Biotech (U.K.), and Aapptec and were used as received unless stated otherwise.

In brief, at the beginning of the synthesis, the resin was allowed to swell in DMF for 20 min. To remove the Fmoc protection from the resin, as well as from subsequent amino acids, a solution of 20% piperidine/DMF (110–89–4, Sigma-Aldrich) was used, and the mixture was shaken for 2 and 7 min, with intermediate aspiration of the solution and a wash with DMF. The amino acids were sequentially incorporated into the resin using dry DMF, dichloromethane (DCM) (75–09–2, Sigma-Aldrich) or *N*-Methyl-2-pyrrolidone (872–50–4, Sigma-Aldrich). For every addition, the appropriate amount of the Fmoc-amino acid (3 equiv) and OxymaPure were dissolved in a minimal amount of DMF (0.5 mL/100 mg resin or 0.2M) and subjected to vortexing for homogenization. Subsequently, DIC was added to the solution, which was then combined with the pretreated resin. The resulting mixture was allowed to react to result in the double-coupling of each amino acid for at least 30 min per coupling under mechanical stirring (1000 rpm). Following the final coupling and deprotection, the resin was washed three times with DMF and once with DCM, and subsequently dried under vacuum. Both arms were then cleaved from the solid support using trifluoroacetic acid (TFA) (76–05–1, Sigma-Aldrich), distilled water and triisopropyl silane (TIPS) (6485–79–6, Sigma-Aldrich) at a ratio of TFA/H_2_O/TIPS 95:2.5:2.5 for 2 h and purified by High Performance Liquid Chromatography (HPLC) followed by characterization with Mass Spectrometry (MS) analysis. Complete coupling was determined with the Kaiser test in all cases (Section S1.1)

Specifically for the 5-FAM-bearing arm, Fmoc-L-Aha–OH (942518–20–9, Iris Biotech) was used to introduce azide moieties, with subsequent Fmoc removal by piperidine and Boc protection for hydrazine stability. The *N*-terminal Boc protection was performed by the addition of di*tert*-butyl decarbonate ((Boc)_2_O) (24424–99–5, Sigma-Aldrich) (5 equiv) and DIPEA (3 equiv) in anhydrous DCM, under 1000 rpm agitation at room temperature for 1 h twice, with an intermediate DCM wash. The product was further purified by three DCM washes and one DMF wash.

#### 3.2.1 Hydrazine Treatment Optimization

The IvDde-protected lysine (204777–78–6, Aapptec) was selectively deprotected using hydrazine solutions (302–01–2, Sigma-Aldrich) under various conditions to optimize efficiency and minimize side reactions, such as arginine ornithination. Initial trials involved a 2% hydrazine solution in DMF applied through the resin for 10 min at a ratio of 12.5 or 75 mL per gram of peptide–resin with agitation at 1000 rpm, followed by washing steps (3 × DCM and 3 × DMF). In the following trials, a 4% hydrazine solution was tested under reduced agitation (300 rpm) at room temperature, using a ratio of 75 mL per gram of peptide–resin. Further optimization trials employed 4% hydrazine at a low temperature (8 °C) (either ×1 or ×3) to moderate reaction kinetics and inhibit arginine ornithination, followed by washing steps (3 × DCM and 3 × DMF) under minimal agitation. MS analyses were performed to monitor the presence of ornithine byproducts resulting from lysine/arginine conversion. Limited hydrazine volume and exposure duration were implemented to minimize side reactions.

#### 3.2.2 Optimisation of FAM Coupling

The fluorophore 5-carboxyfluorescein (5-FAM) (76823–03–5, Aapptec) was coupled using DIC and OxymaPure as activators. Standard conditions (3:3:3 molar ratio of FAM/DIC/OxymaPure) were initially employed, but alternative ratios were tested to mitigate byproduct formation. These included 1:1:1, 0.5:4:2 and 2:4.5:4.5 molar ratios of FAM:DIC:OxymaPure. Reaction times varied from 1 to 2 h, and the impact of acidic versus basic environments, deploying PyBOP (128625–52–5, Aapptec) and *N*,*N*-Diisopropylethylamine (DIPEA) (7087–68–5, Sigma-Aldrich) at 3:6 mol equiv. Sequential coupling strategies were tested: (1) FAM addition before azide and (2) azide addition prior to FAM. DMol^3^ simulations calculated activation energies (*E*_A_) and reaction energies (*E*) for all possible products in both coupling scenarios. The energies of the desired product and byproducts were compared to determine reaction feasibility and kinetics. HPLC analysis was performed to quantify yields and identify byproducts.

### 3.3 Peptide Characterization

Each peptide–resin complex was mixed with the scavenger solution consisting of trifluoroacetic acid, triisopropylsilane and DI water (95% TFA: 2.5% TIS: 2.5% H_2_O) (v/v) (100 μL/10 mg product) to remove all the side chain protective groups and separate the peptide from the resin. The resulting mixture in all cases was allowed to react for at least 2 h under mechanical stirring (1000 rpm). Then, the resin was filtered from the synthesis product, and the solvents were evaporated under an inert nitrogen atmosphere. Subsequently, chilled Diisopropyl ether (DIPE) (108–20–3, Sigma-Aldrich) was added to the mixture, and it was centrifuged for 3 min at 5000 rpm for sample-based and 15 min at 4000 rpm at 0 °C for large-scale deprotection of the final product. DIPE, as an organic solvent, aids in the precipitation of the peptide by promoting its separation from the solvent and impurities, thereby enhancing product purity. The supernatant was aspirated, and the peptide pellet was dissolved in acetonitrile (75–05–8, VWR) and HPLC-grade water (7732–18–5, VWR), CH_3_CN-H_2_O (1:1). Subsequently, it was suitably diluted and characterized to determine its purity and synthesis efficiency. Analytical HPLC was performed on the Shimadzu LC20 system using LabSolutions software (v.5.92) for data processing per the experimental process outlined in Section S1.2.

### 3.4 Purification

Preparative and semipreparative HPLC was employed to purify the individual arms (FAM-bearing and Dabcyl-bearing arms) and the beacon. A preparative column (Gemini 5 μm NX-C18 110A, 250 × 21.2 mm, Phenomenex) with a scalability factor of 48.8 and a semipreparative column (Gemini 5 μm NX-C_18_ 110A, 250 × 10 mm) with a scalability factor of 10.9 were used. The HPLC system comprised a Gilson GX-271 unit equipped with a 159 UV–vis detector, a 322 pump, and Triluition LC software. The mobile phases consisted of 0.1% trifluoroacetic acid (TFA) in water (Phase A) and 0.1% TFA in acetonitrile (Phase B). Two gradient elution methods were evaluated per Section S1.3, and all peaks were collected and analyzed with the purified products being freeze-dried for further analysis and applications.

### 3.5 Bioconjugation via Copper­(I)-Catalyzed Azide–Alkyne Cycloaddition (CuAAC)

Individually synthesized and purified peptide arms were conjugated using copper-catalyzed azide–alkyne cycloaddition (CuAAC). Stock solutions of CuSO_4_ (20 mM), THPTA (50 mM), sodium ascorbate (100 mM), and aminoguanidine hydrochloride (100 mM) were prepared in ultrapure water. Reaction conditions were optimized to 0.1 mM CuSO_4_, 0.5 mM THPTA, and 5 mM each of sodium ascorbate and aminoguanidine HCl in deoxygenated 0.1 M phosphate buffer (pH 7.0). In a total volume of 500 μL, the azide- and alkyne-bearing peptide arms (in DMF) were combined at a 1.2:1 molar ratio, followed by sequential addition of a premixed CuSO_4_/THPTA catalyst (preincubated for 30 min), sodium ascorbate, and aminoguanidine HCl. The reaction was conducted under a nitrogen atmosphere in the dark at 45 °C for 4 h with constant stirring. Detailed experimental conditions are provided in Section S1.4.

### 3.6 Peptide Sensing for S100B Detection

Initial spectral characterization of the beacon was conducted to determine optimal emission wavelengths using a microplate reader (Varioskan LUX Multimode, Thermo Fisher). Fluorescence emission spectra were recorded from 517 to 620 nm using an excitation wavelength of 499 nm. All experiments were performed in binding buffer containing 50 mM Tris-HCl buffer (pH 7.0 at the experimental temperature, unless stated otherwise), supplemented with 240 mM NaCl and 20 mM CaCl_2_ (Sigma-Aldrich).^58,59^ Preliminary binding affinity experiments were conducted at three temperatures: ambient 25, 30, and 35 °C.^46^ Analysis indicated enhanced signal acquisition at 35 °C compared to lower temperatures; thus, all subsequent measurements were performed at 35 °C. A 100 μM stock solution of the beacon was prepared in the experimental buffer. To evaluate the effect of pH on the fluorescence response of the probe, emission spectra were recorded across a pH range of 6.0–8.0 in the presence of S100B.^60^ The stock binding buffer was divided into aliquots, and the pH of each was adjusted to 6.0, 6.5, 7.0, 7.5, and 8.0 using minimal volumes of concentrated HCl or NaOH (1.0 M) to maintain consistent ionic strength across all conditions. Fluorescence emission spectra were collected at 35 °C for solutions containing a fixed concentration of bioreceptor (500 nM), as well as at four different concentrations of S100B (0, 5, 20, and 100 nM) (abx060130, Abbexa), to assess the pH-dependence of sensor performance. Binding assays were performed with varying concentrations of S100B protein (0, 0.05, 0.5, 5, 10, 25, 50, 75, and 100 nM) while maintaining a constant bioreceptor concentration of 1 μM or 500 nM. Protein aliquots were stored on ice to prevent denaturation. Before mixing with the bioreceptor, protein solutions were preincubated in the buffer for 10 min to facilitate structural equilibration and optimal binding site exposure. Samples were then incubated at 35 °C for 10 min before the initial fluorescence measurement. Subsequent measurements were recorded following a 30 min incubation at 35 °C under minimal low-force shaking (180 rpm) for up to 90 min. Assays were conducted in black bottom 96-well plates, and each condition was tested in triplicate across a minimum of three independent experiments. Negative controls included (i) a no-protein control and (ii) a blank binding buffer control to establish baseline fluorescence and ensure accurate background subtraction.

Human serum (Sigma-Aldrich, H4522) was diluted 1:10 (v/v) in assay buffer and spiked with S100B at 0, 0.5, 5, and 10 nM, covering the diagnostic range. All samples were incubated for 60 min at 35 °C and quantified using the S100B sensing system. Observed signals were converted to concentrations using the 60 min standard curve, and mean recovery was calculated as the percentage of the observed concentration relative to the expected spike.

#### 3.6.1 Safety Statement

Dichloromethane (DCM), N,N-dimethylformamide (DMF), piperidine, Oxyma Pure, and trifluoroacetic acid (TFA) are hazardous and were handled with appropriate precautions. DCM is volatile and a suspected carcinogen; DMF is toxic by inhalation or skin contact; piperidine and TFA are corrosive; and Oxyma Pure may cause respiratory sensitization. All reagents were used in a certified fume hood with suitable PPE (lab coat, safety goggles, and chemical-resistant gloves). Waste was disposed of in accordance with institutional and regulatory guidelines.

#### 3.6.2 Ethical Statement

Human serum samples used in this study were obtained commercially from Sigma-Aldrich, a verified supplier which guarantees that all samples were collected under appropriate informed consent and according to institutional ethical guidelines. Thus, all biological material was obtained following the supplier’s compliance with institutional review and donor consent protocols.

## 4 Conclusion

Early detection and monitoring of tumor biomarkers are critical for improving cancer management and patient outcomes.^61^ In this work, an optical biosensing probe was designed and developed for the single-step sensitive detection of S100B, a diagnostic and prognostic biomarker for melanoma skin cancer. By leveraging FRET and PNA interactions, our system achieves a subnanomolar detection limit (∼0.045 nM) and enables fast quantification within 60 min, with superior sensitivity and selectivity, even in complex biological samples like human serum with a mean recovery of ≤108%. Activation energy simulations facilitated engineering of a synthetic route that enables site-specific fluorophore conjugation within the peptide–PNA beacon architecture, overcoming key limitations associated with bifunctional fluorophore integration in SPPS. Our approach facilitated the incorporation of 5-FAM into the complex TRTK12-containing probe, preserving biorecognition capabilities while ensuring efficient FRET signaling.

The potential of using our fluorescent probe was demonstrated for skin cancer detection by targeting S100B, laying the groundwork for integration with minimally invasive sampling solutions such as microneedle patches or hydrogel-based ISF collection systems.^62,63^ Peptides are particularly useful in these applications because their small size and synthetic nature allow for better stability, easier functionalization, and faster binding kinetics. Furthermore, the assay’s compatibility with physiological temperature conditions supports its potential integration into transdermal devices.

Overall, our results highlight the promise of combining advanced molecular design with engineered synthesis routes and optical biosensing technology to improve detection of melanoma biomarker S100B, potentially reduce diagnostic delays and the reliance on biopsy, and ultimately enhance clinical decision-making in oncology.

## Supplementary Material



## Data Availability

The data supporting this article have been included as part of the Supporting Information.

## Notes

Comprehensive experimental procedures and supplementary results, including chemical structures and chromatograms (PDF)
