# Supplementary material for: Peptide-Based Fluorescent Biosensing System for the Detection of the Melanoma Biomarker S100B
Source: Bioconjug Chem. 2025 Nov 6;36(11):2357–69. doi: 10.1021/acs.bioconjchem.5c00337 (PMC12635975; doi:10.1021/acs.bioconjchem.5c00337)
Supplement: Supplementary file 1 [file bc5c00337_si_001.pdf]

Supplementary Information

Peptide-Based Fluorescent Biosensing System for the Detection of the Melanoma Biomarker S100B

Eleni Chatzilakou[a,*], Yubing Hu[a,b], Othman Al Musaimi[a, c, d], Lucia Lombardi[a, e], Oscar M. Mercado-Valenzo[a, d], Nan Jiang[f], Daryl R. Williams[a, d], Ali K. Yetisen[a,*]

[a.] Department of Chemical Engineering, Imperial College London, South Kensington, London, SW7 2BU, United Kingdom

[b.] Centre for AIE Research, Guangdong Provincial Key Laboratory of New Energy Materials Service Safety, College of Materials Science and Engineering, Shenzhen University, Shenzhen, 518060 China

[c.] Faculty of Medical Sciences - School of Pharmacy, Newcastle University, Newcastle upon Tyne, NE17RU, United Kingdom

[d.] Orthogonal Peptides Limited, London, SW7 2AZ, UK, United Kingdom

[e.] School of Biological Sciences, Queen's University Belfast, 19 Chlorine Gardens, Belfast, BT9 5DL, United Kingdom

[f.] West China School of Basic Medical Sciences & Forensic Medicine, Sichuan University, Chengdu 610041, China

***Corresponding authors:** e.chatzilakou22@imperial.ac.uk, a.yetisen@imperial.ac.uk

1. Experimental Section

1.1 Synthesis and Optimisation of the Bioreceptor

Coupling efficiency was evaluated using the Kaiser test, a qualitative assay based on the ninhydrin reaction. A 0.2% (w/v) ninhydrin (485-47-2, Sigma Aldrich) solution in ethanol was prepared and stored in a glass vial. Following each amino acid or chemical moiety addition, a small quantity of resin was thoroughly dried and transferred to a glass vial containing 200 µL of ninhydrin solution. The mixture was then heated at 110°C for 5 minutes. A blue or purple colouration indicated the presence of free primary amines, signifying incomplete coupling, whereas a yellow or colourless result confirmed successful coupling. To validate assay reliability, a control resin, amino C6 methacrylate (LS01375-186, Purolite, Fischer Scientific), was tested in parallel with experimental samples. The Kaiser test was performed after each coupling step to ensure a complete reaction before proceeding to the next synthetic stage.

1.2 Peptide Characterisation

The LC column used was a Symmetry Luna C_{18} (3.6 µm, 4.6 × 150 mm), with a flow rate of 1.0 mL/min and UV detection at 280 nm. The mobile phase A was 0.1% TFA in H_2O, and mobile phase B was 0.1% TFA in CH_3CN at 20 °C. HPLC analysis was performed using two distinct gradient elution methods to optimise analyte separation. The first method, referred to as the 5–70% of mobile phase B in 15 min gradient, was initiated with 5% mobile phase B, maintained for 3 minutes, followed by a linear increase to 70% at 18 minutes. The concentration was then ramped to 95% at 20 minutes and held until 23 minutes. At 25 minutes, mobile phase B was returned to 5% and maintained until 35 minutes to ensure complete column re-equilibration. The second method, the 5–95% B in 15 min gradient, began with 5% Solvent B, which was linearly increased to 95% at 16 minutes and maintained until 20 minutes. At 21 minutes, Solvent B was returned to 5% and held until 25 minutes for re-equilibration. For both methods, the system was pre-equilibrated with the initial solvent composition before each injection. Chromatographic results are presented exclusively for the primary gradient elution method. Mass spectrometry (MS) was performed using a Velos Pro mass spectrometer (ThermoFisher Scientific, Waltham, MA, USA), a hybrid linear trap quadrupole (LTQ)-Orbitrap system, operated in positive electrospray ionisation mode (ESI+-MS). Samples were analysed either by direct infusion or liquid chromatography (LC)-MS, depending on the experimental setup. The LC setup included a Dionex Ultimate 3000 HPLC system from ThermoFisher Scientific with 20 mm L x 0.075 mm ID and 150 mm x 0.075 mm ID PepMap 100 C18 trap and analytical columns packed with 3 µm particles to concentrate and separate the sample, respectively. The typical LC gradient involved an initial loading phase of the sample into the trap column by the loading pump at 5 µL/min in 3% solvent B (0.1% TFA in acetonitrile) for 5 minutes. The line was then switched such that the nanocapillary pump flowed at 0.5 µL/min through both the trap and analytical column in sequence to the mass spectrometer. Simultaneously, the

mobile phase gradient increase from 3% to 80% Acetonitrile in 40 minutes, was initiated. The composition was maintained at 80% for 10 minutes before returning to 3% over 5 minutes, with an additional equilibration period at 3% for 5 minutes.

1.3 Purification

The linear gradient method involved an initial isocratic phase of 3 minutes at 5% B, followed by a gradient phase lasting 15, 30, or 60 minutes from 5% to 70% B at rates of 4.33%, 2.16%, or 1.08% per minute, respectively, and concluding with a final isocratic phase at 70% of mobile phase B and a wash phase of 5 minutes at 5% of mobile phase B. The ramp method similarly began with an isocratic phase of 3 minutes at 5% B, followed by a gradient phase divided into three stages: 5 minutes from 5% to 25% of mobile phase B at 4% per minute, 15 minutes from 25% to 40% of mobile phase B at 1% per minute, and 10 minutes from 40% to 70% of mobile phase B at 3% per minute. This was followed by a final isocratic phase at 70% B and a 5-minute wash phase at 5% of mobile phase B.

Flow rates ranged from 2 mL/min to 10 mL/min, with sample injection concentrations varying between 2 mg/mL and 10 mg/mL. The scalability factor was initially determined from empty column volumes but was subsequently adjusted based on the pressure limitations of the instrument and columns. The selected linear gradient method, involving a 60-minute gradient at 1% of mobile phase B per minute, was applied to purify the individual arms using a 10 mg/mL sample injection and a 10 mL/min flow rate. For the semi-preparative setup, purification of the beacon was achieved with a 5 mg sample injection in 1 mL and a flow rate of 4 mL/min, using a gradient from 5% to 70% of mobile phase B over 60 minutes.

All samples were thoroughly dried and freeze-dried after being immersed in liquid nitrogen to ensure proper storage before subsequent use.

1.4 Bioconjugation via Copper(I)-catalysed Azide-Alkyne Cycloaddition (CuAAC)

Individually synthesised and purified peptide arms were conjugated via copper-catalysed azide-alkyne cycloaddition (CuAAC) to form the target peptide beacon. Stock solutions were prepared in ultrapure water as follows: copper(II) sulfate pentahydrate (7758-99-8, Sigma Aldrich) at 20 mM, tris(3-hydroxypropyltriazolylmethyl)amine (THPTA) (760952-88-3, Broadpharm) (ligand) at 50 mM, sodium ascorbate (134-03-2, Sigma Aldrich) at 100 mM, and aminoguanidine hydrochloride (A.HCl) (1937-19-5, Sigma Aldrich) at 100 mM. The final reaction conditions were adjusted to achieve concentrations of 0.1 mM (Cu), 0.5 mM (ligand), and 5 mM each for sodium ascorbate and A.HCl. Before reaction assembly, dissolved oxygen was removed from the phosphate buffer (pH 7.0, 0.1M of KH_2PO_4 and K_2HPO_4) by purging with N_2 and sonicating. The reaction tube was also purged with N_2 both before and after reagent addition. In a 500 µL reaction volume (conducted in a 1.5 mL Eppendorf tube), the following components were added in the specified order to maintain consistent ionic strength and pH: 12.5 µL of the azide-bearing arm (in DMF) and 12.5 µL of the alkyne-bearing arm (in DMF), for a total of 25 µL of biomolecules; 417 µL of phosphate buffer (95:5 buffer/DMF v/v was used as the reaction solvent); 7.5 µL of a premixed solution of $CuSO_4$ and THPTA (2.5 µL $CuSO_4$ and 5 µL THPTA), which was allowed to pre-react under minimal agitation for 30 min; 25 µL of A.HCl, and 25 µL of sodium ascorbate. The azide-bearing arm was used at a 1.2 molar ratio relative to the alkyne-bearing arm, corresponding to final concentrations of 400 µM and 333 µM, respectively, to minimise dimerisation and aggregation of the alkyne-functionalized (dabcyl-bearing) arm. The reaction mixture was stirred at 500 rpm under a nitrogen-inert atmosphere and in the dark at 45°C for 4 hours. The use of a 500 µL working volume in a 1.5 mL tube (or 5 mL in a 15 mL Falcon tube) ensured adequate mixing and headspace during the reaction.

2 Results and Discussion

2.1 Design and Computational Simulation of Peptide Sequence

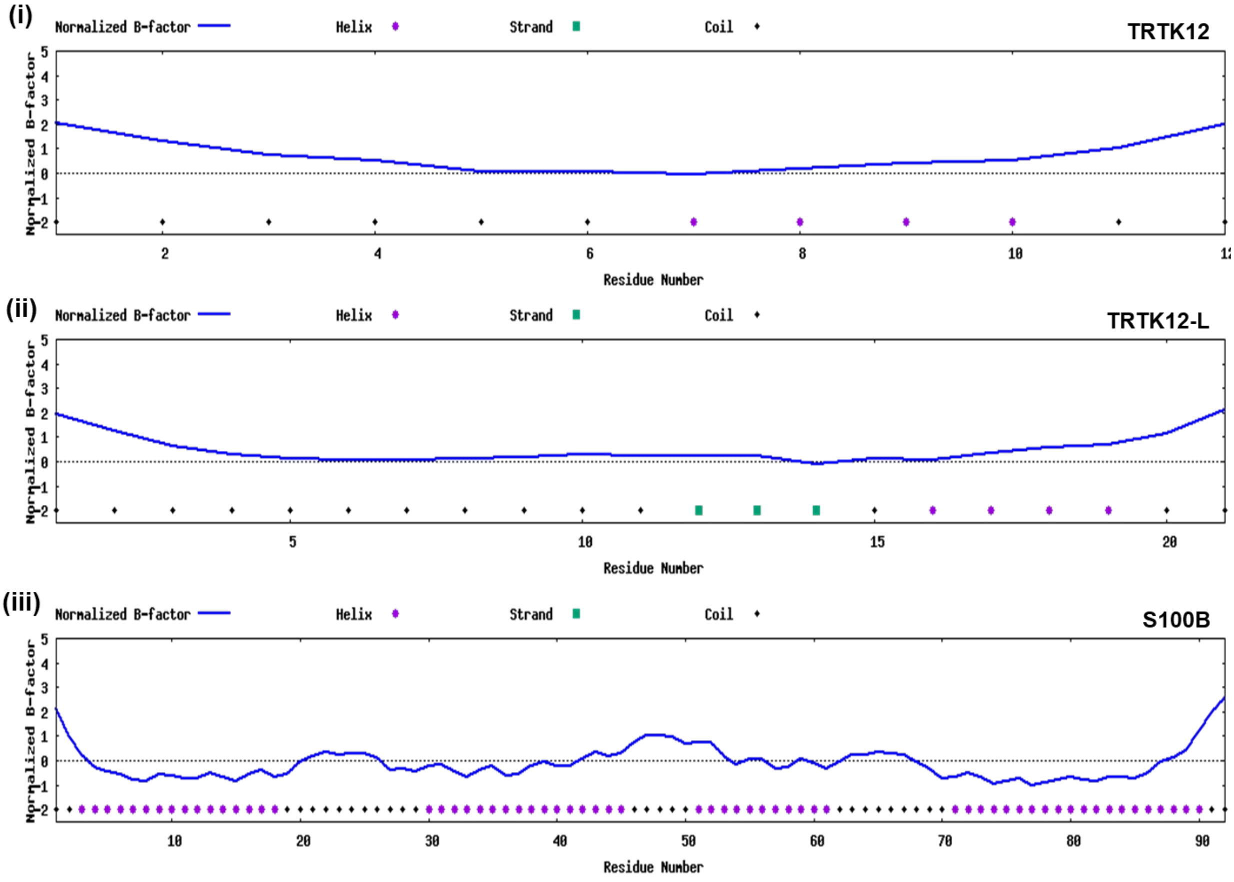

Figure S1. The normalised B-factor (called B-factor profile, BFP) of (i) TRTK12, (ii) TRTK12-L and (iii) S100B.

Table S1.Ramachandran analysis of TRTK12 peptide residues with φ, ψ, and ζ angles of residues in the TRTK12 peptide sequence (TRTKIDWNKILS), categorised by structural type (Loop or Helix).

Residue	Type	Φ	Ψ	Z
ARG	Loop	-107.03	3.31	-0.2188
THR	Loop	-59.77	-169.6	-1.576
LYS	Loop	-123.39	32.98	-0.9189
ILE	Loop	50.37	-16.67	-1.4432
ASP	Helix	31.11	88.69	-1.0258
TRP	Helix	-35.71	-48.89	-1.1982
ASN	Helix	-81.39	8.4	-0.8893
LYS	Helix	-116.37	-37.05	-1.1655
ILE	Helix	-62.3	-33.19	-1.0058
LEU	Helix	-67.48	-2.44	-1.2479

Table S2. Ramachandran analysis of TRTK12-L peptide residues with φ, ψ, and ζ angles of residues in the TRTK12-L peptide sequence (GGRRRRGLGTRTKIDWNKILS), categorised by structural type (Loop or Helix).

Residue	Type	Φ	Ψ	Z
GLY	Loop	73.51	-50.09	-0.8248
ARG	Loop	-49.73	-63.07	-1.1692
ARG	Loop	-62.91	-27.4	2.8745
ARG	Loop	-62.16	-19.53	2.7505
ARG	Loop	-66.64	-8.32	-0.1075
GLY	Loop	-62.25	-10.51	-0.6343
LYS	Loop	71.99	152.45	-1.1038
GLY	Loop	-55.65	-32.93	-0.2191
THR	Loop	-61.31	-0.55	-1.576
ARG	Loop	72.42	-34.15	-1.1948
THR	Loop	-53	119.06	-1.3137
LYS	Loop	-62.65	17.96	-1.1038
ILE	Loop	-42.87	126.01	-1.4125
ASP	Helix	-72.6	130.31	-1.0078
TRP	Helix	-59.04	-39.86	0.1076
ASN	Helix	-66.37	-9.98	-0.8707
LYS	Helix	-116.75	-8.07	-1.1626
ILE	Helix	-110.76	-13.72	-1.27
LEU	Helix	-53.59	-13.77	-1.2509

Table S3. HADDOCK analysis results for TRTK12 and S100B in monomeric form, representing mean values.

Cluster No	HADDOCK score	Cluster size	RMSD from the overall lowest-energy structure	Van der Waals energy	Electrostatic energy	Desolvation energy	Restraints violation energy	Buried surface area	Z-score
2	-77.9	25	6.7	-27.4	-184.5	-18.8	51.1	1043.5	-1.5
9	-69.9	5	5.0	-33.1	-128.3	-15.4	42.7	1200.1	-0.7
7	-69.7	7	7.6	-32.4	-77.5	-23.4	16.1	1032.0	-0.7
8	-66.9	7	5.2	-35.5	-123.0	-11.5	48.2	1043.9	-0.4
1	-65.9	28	7.7	-37.7	-76.7	-17.2	43.0	1098.9	-0.3
3	-64.1	17	6.1	-27.8	-119.4	-18.7	62.8	1253.2	-0.1
4	-60.6	10	7.8	-27.4	-112.9	-18.4	77.7	991.8	0.2
5	-60.4	9	7.8	-31.4	-72.5	-17.0	25.5	928.9	0.2
6	-50.1	8	6.2	-23.3	-93.5	-16.4	83.4	1025.0	1.3
10	-42.0	4	5.8	-21.9	-51.5	-15.1	54.2	931.9	2.1

Table S4. HADDOCK analysis results for TRTK12-L and S100B in monomeric form, representing mean values.

Cluster No	HADDOCK score	Cluster size	RMSD from the overall lowest-energy structure	Van der Waals energy	Electrostatic energy	Desolvation energy	Restraints violation energy	Buried surface area	Z-score
7	-91.4	6	5.7	-31.7	-309.4	1.8	3.9	1310.9	-1.4
4	-87.8	9	8.9	-51.5	-137.8	-15.3	65.2	1436.1	-1.1
5	-84.5	6	6.1	-29.7	-285.4	-2.1	43.2	1299.2	-0.8
2	-84.4	18	5.9	-29.9	-248.9	-9.6	49.9	1202.0	-0.8
9	-78.1	5	1.0	-32.2	-235.8	-4.9	62.0	1284.0	-0.2
3	-75.3	12	9.5	-33.8	-154.3	-17.3	66.8	1257.4	0
12	-72.3	4	9.8	-35.4	-135.8	-15.9	60.9	1317.0	0.3
1	-68.0	41	9.5	-29.6	-160.6	-8.4	21.1	1092.6	0.7
8	-60.5	5	5.6	-19.7	-193.8	-4.7	26.0	911.7	1.3
10	-53.5	4	10.3	-36.3	-85.9	-9.7	96.4	1175.1	1.9

2.2 Synthesis and Optimisation of the Bioreceptor

2.2.1 Optimisation of IvDde removal protocol

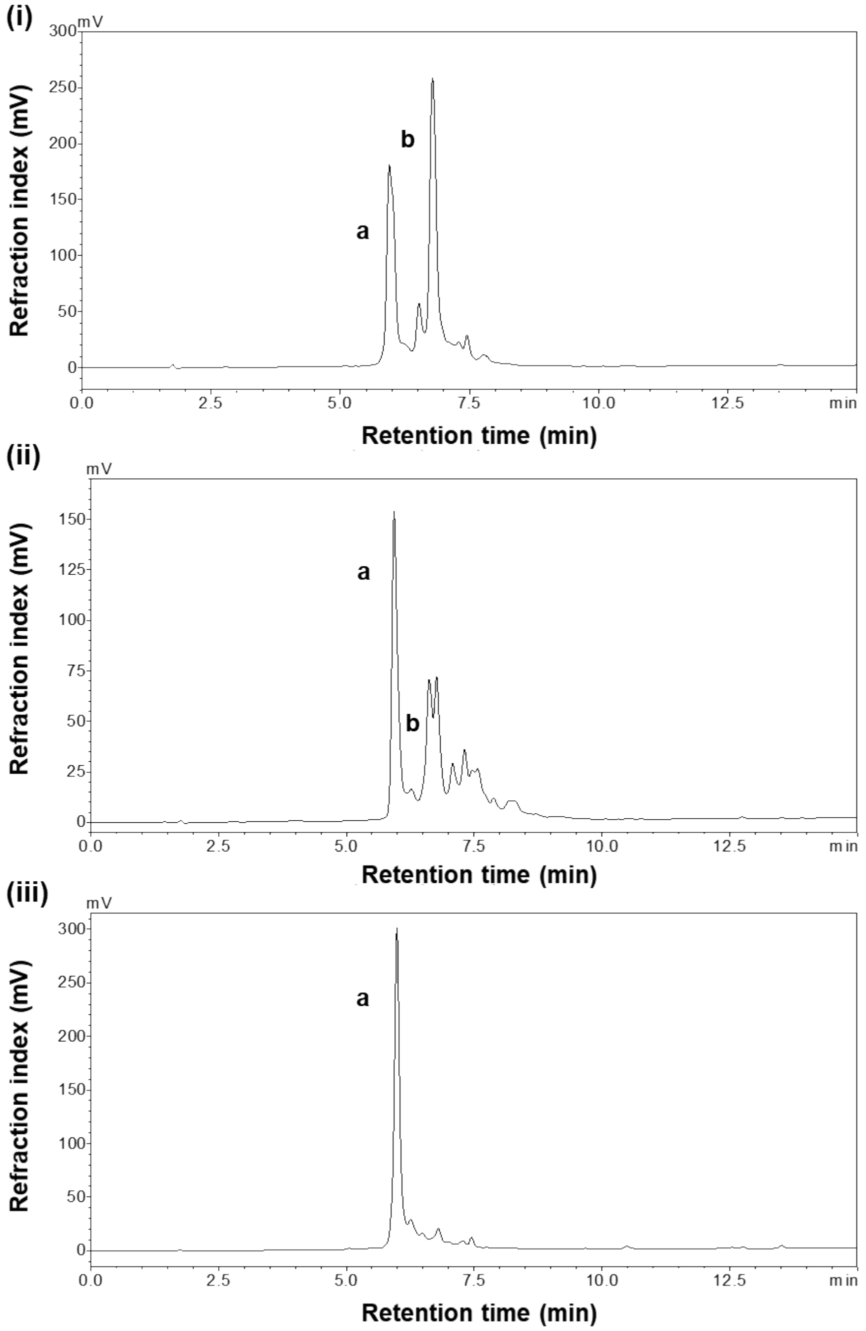

Figure S2. HPLC chromatograms of the FAM-arm synthesis product following hydrazine treatment at room temperature: (i) 2% hydrazine (1× for 10 min) in 12.5 mL per gram of resin-product, (ii) 2% hydrazine (3× for 3 min) in 75 mL per gram of resin-product, and (iii) 4% hydrazine (2× for 3 min, 1× for 5 min) in 75 mL per gram of resin-product. Label (a) represents the fully deprotected product, while label (b) denotes the product containing IvDde.

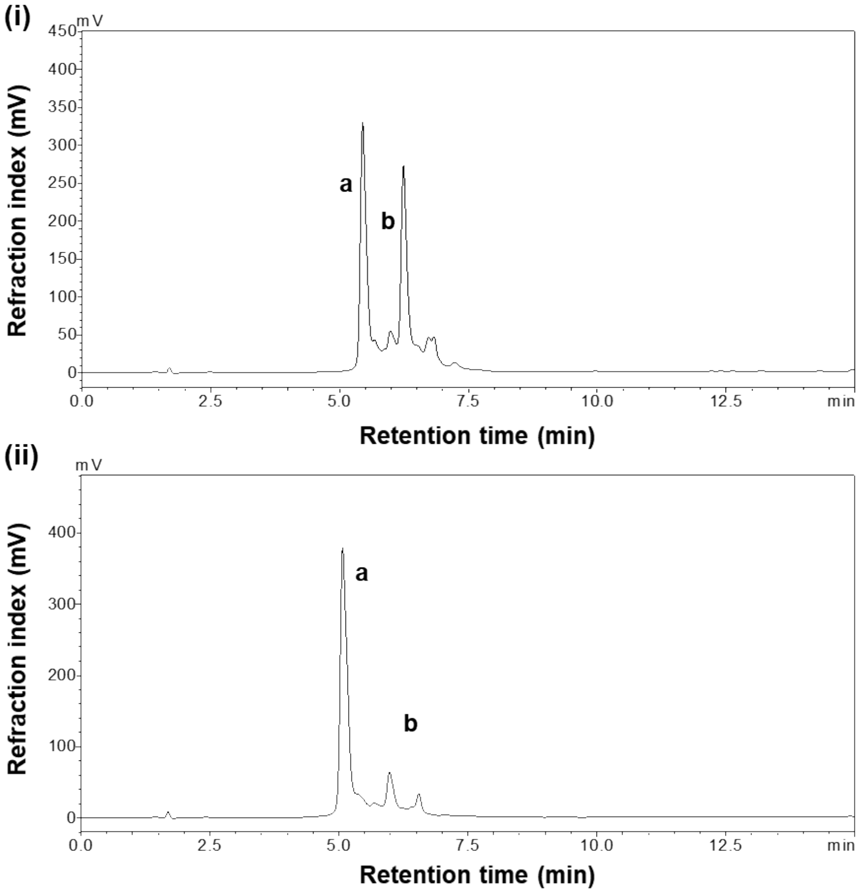

Figure S3. HPLC chromatograms of the FAM-arm synthesis product following hydrazine treatment at 8 °C: (i) 4% hydrazine (1× for 3 min) and (ii) 4% hydrazine (3× for 3 min), in 75 mL per gram of resin-product. Label (a) represents the fully deprotected product, while label (b) denotes the product containing IvDde.

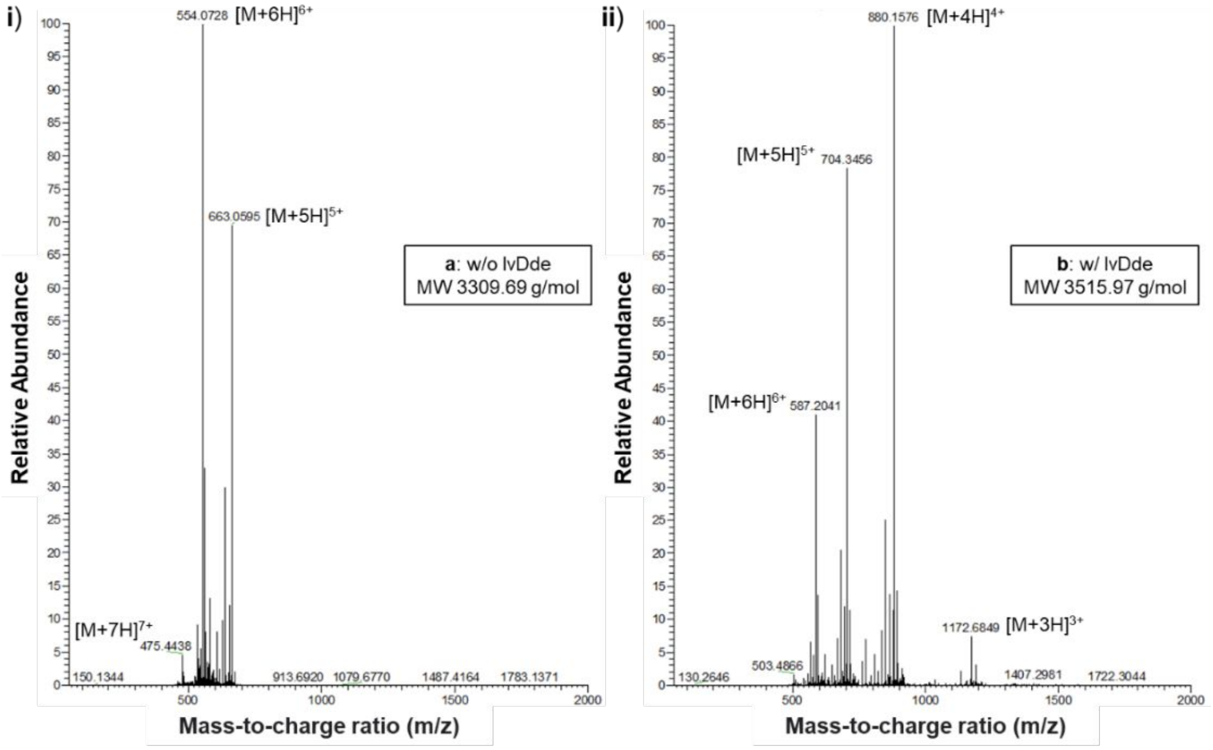

Figure S4. MS analysis of the predominant peaks corresponding to the FAM-arm synthesis product after hydrazine treatment for IvDde deprotection, identifying (i) the fully deprotected product and (ii) the product containing IvDde, with elution times of 6 and 6.6 minutes, respectively.

2.2.2 Optimisation of FAM coupling

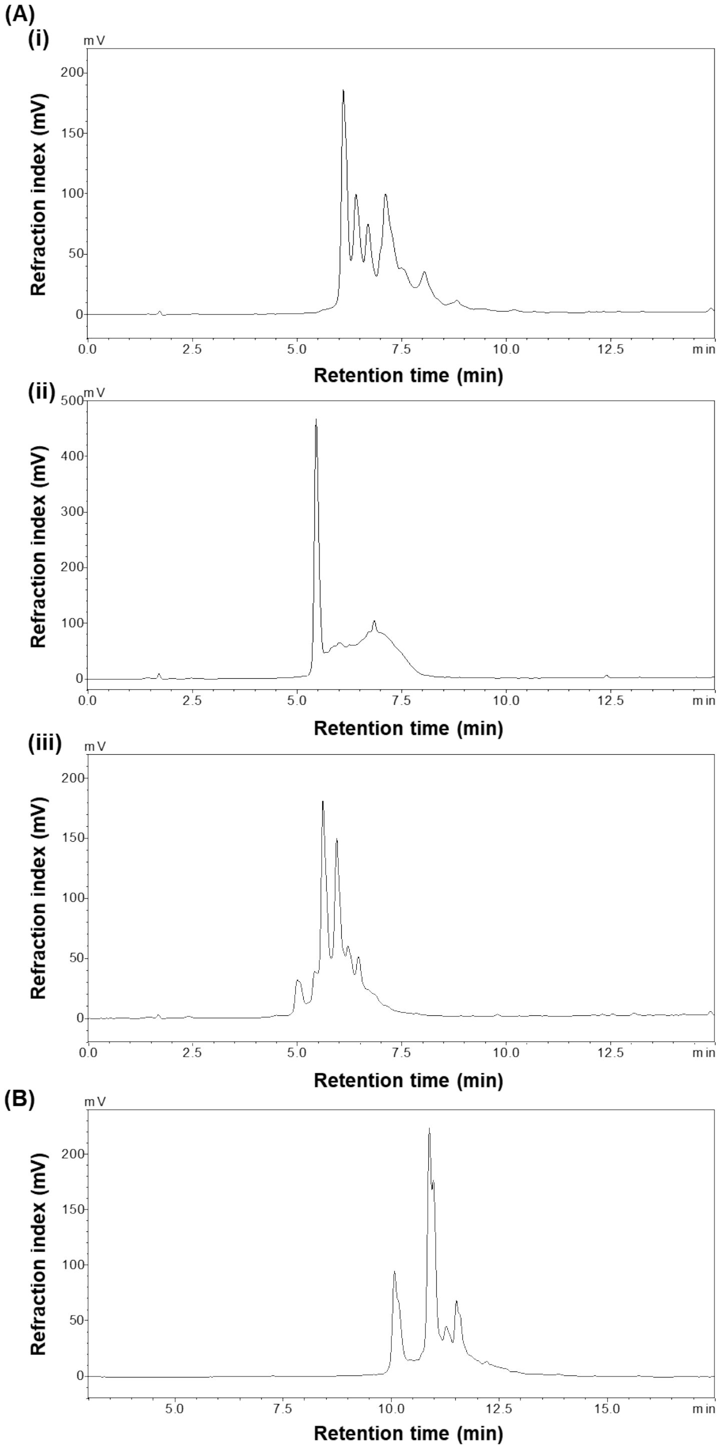

Figure S5. HPLC chromatograms of the FAM-arm synthesis product following the addition of 5-FAM under varying reaction conditions. (A) Depicts the chromatogram obtained using the initial reaction conditions and a 5–95 elution gradient, which resulted in unsuccessful synthesis and necessitated optimisation. Reaction conditions tested included: (i) single coupling with equimolar ratios of 5-FAM:DIC:Oxyma Pure at 3:3:3, (ii) single coupling at 0.5:4:2, and (iii) single coupling at 2:4.5:4.5. (B) Shows the chromatogram obtained under optimised conditions with a 1:1:1 ratio of 5-FAM:DIC:Oxyma Pure using a 5–70 elution gradient

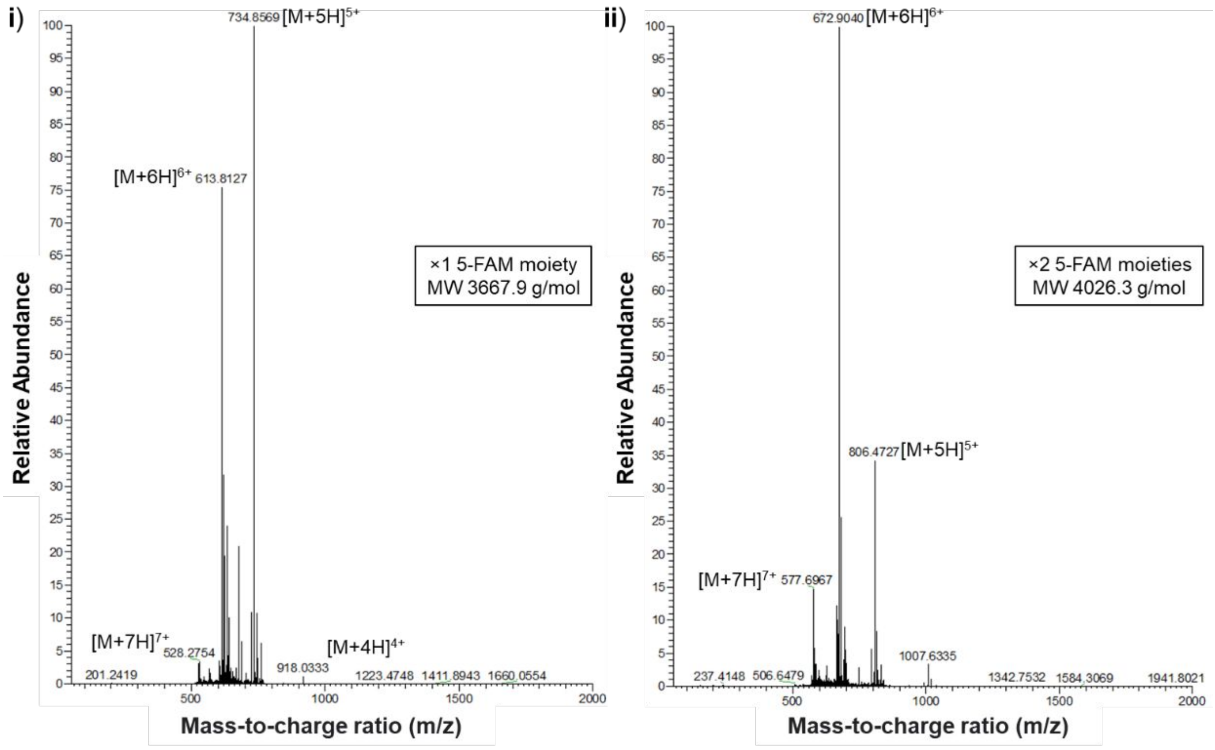

Figure S6. MS analysis of the predominant peaks corresponding to the FAM-arm synthesis product after 5-FAM conjugation was performed. The reaction was conducted using equimolar ratios of 5-FAM, DIC, and Oxyma Pure at (3:3:3), (0.5:4:2), or (2:4.5:4.5), and the results highlight the prevalence of doubly labelled products under conditions with higher equimolar ratios, emphasising the need for re-evaluation of the reaction parameters to optimise product specificity.

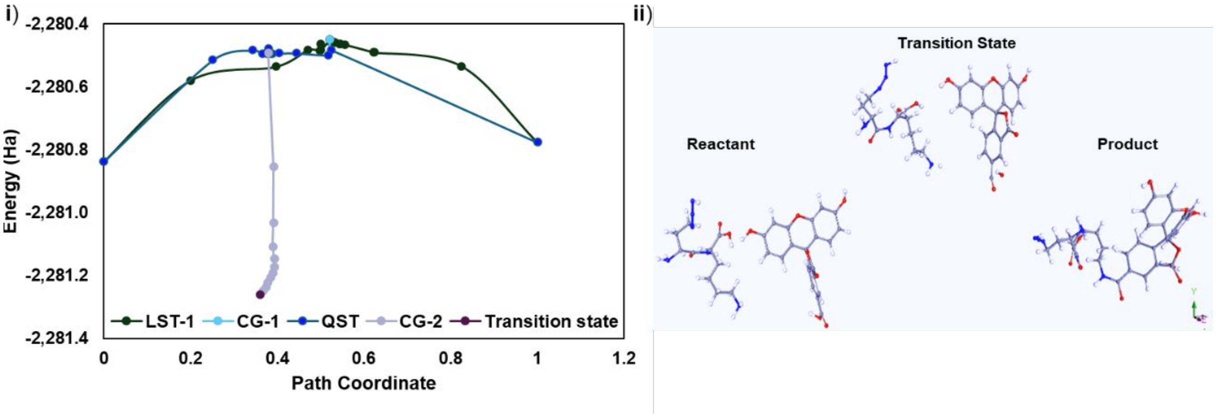

Figure S7. Results of the DMol3 reaction simulation performed in Materials Studio, illustrating potential pathways for the formation of the main product. (i) Depicts the proposed reaction mechanism, while (ii) highlights the simulated transition states of the reactants and products. The analysis incorporates Linear Synchronous Transit (LST), Conjugate Gradient (CG1 and CG2), and Quadratic Synchronous Transit (QST) methods to identify the transition states and validate the reaction pathway.

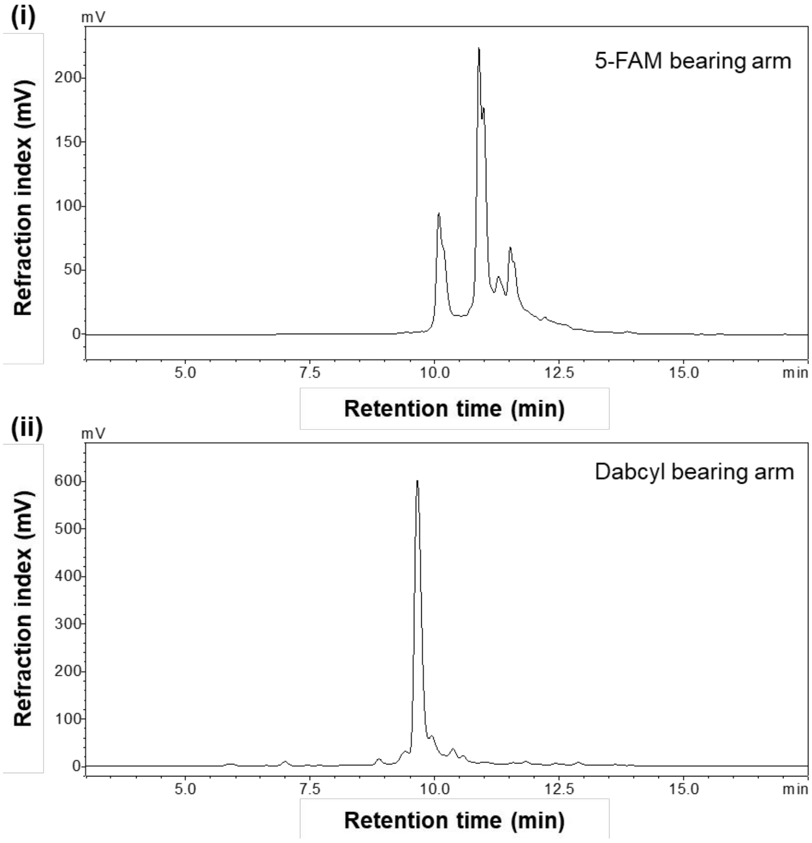

Figure S8. HPLC chromatograms of the crude peptide arms after the completion of individual syntheses are shown, highlighting (i) the 5-FAM-bearing arm and (ii) the Dabcyl-bearing arm. The emphasis on achieving high-yield synthesis aims to reduce purification demands and minimise product loss.

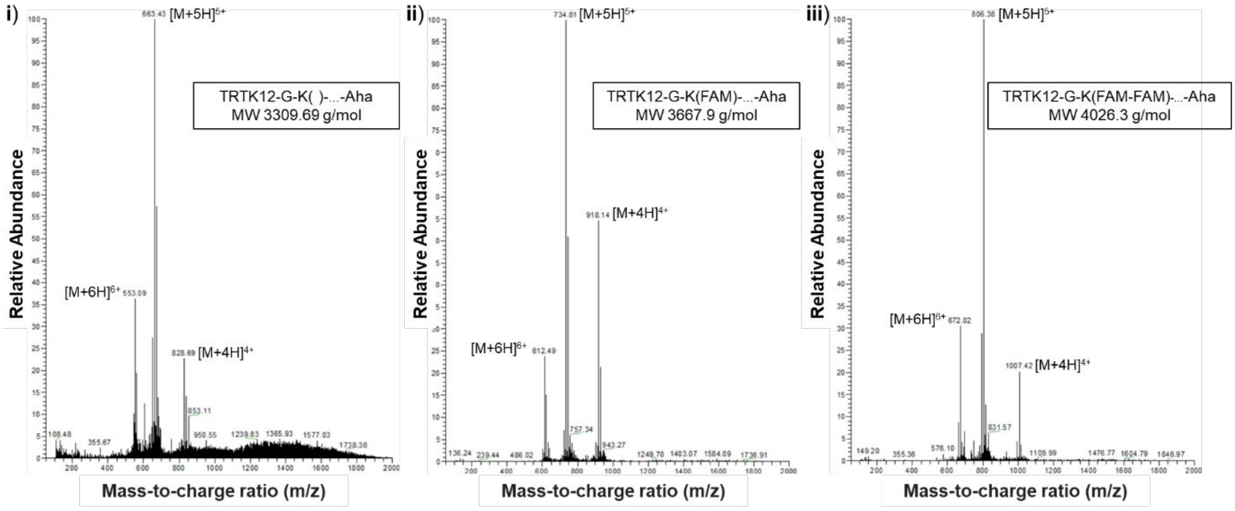

Figure S9. MS analysis of the three chromatographic peaks corresponding to elution times of (i) 10.5 min, (ii) 11.0 min, and (iii) 11.5 min, observed for the primary synthesis product after 5-FAM conjugation. The reaction was conducted using equimolar ratios (1:1:1) of 5-FAM, DIC, and Oxyma Pure.

2.3 Purification

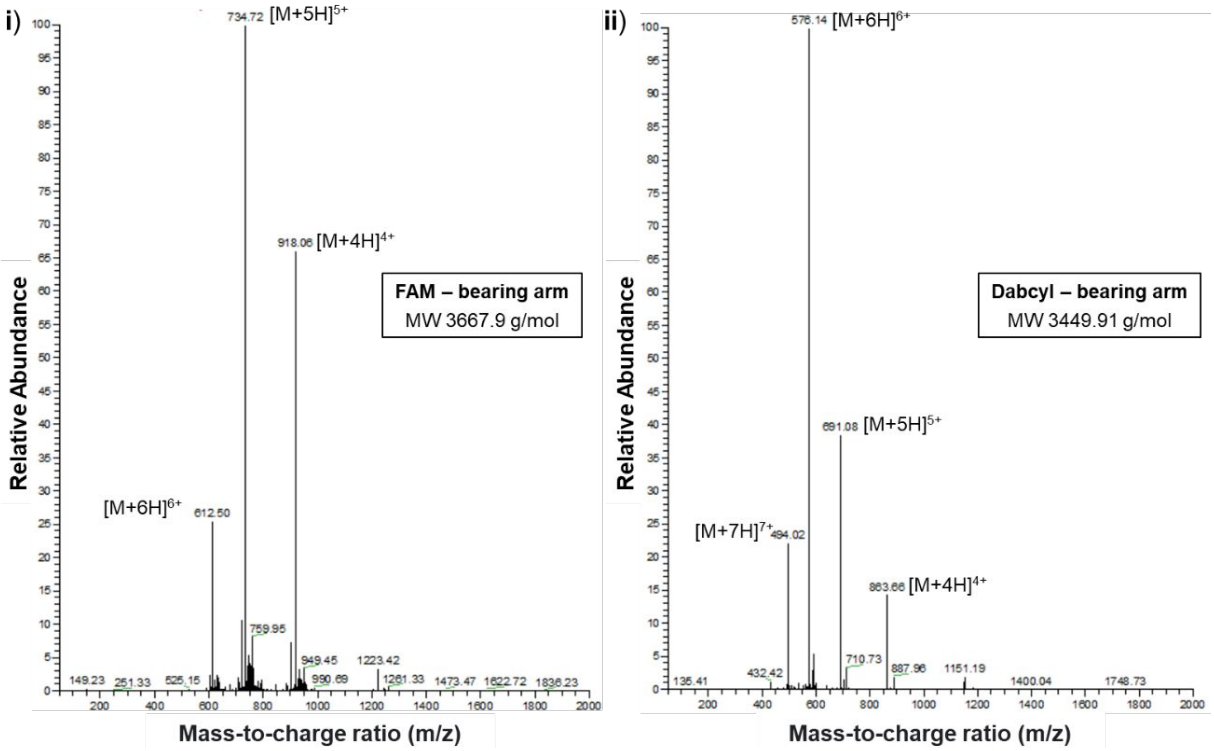

Figure S10. MS analysis results of the key fragments collected from the Gilson during purification confirm the successful synthesis of each peptide arm: (i) the 5-FAM-bearing arm and (ii) the Dabcyl-bearing arm. The purity determined by the relative abundance of the fragments exceeded 90% in both cases.

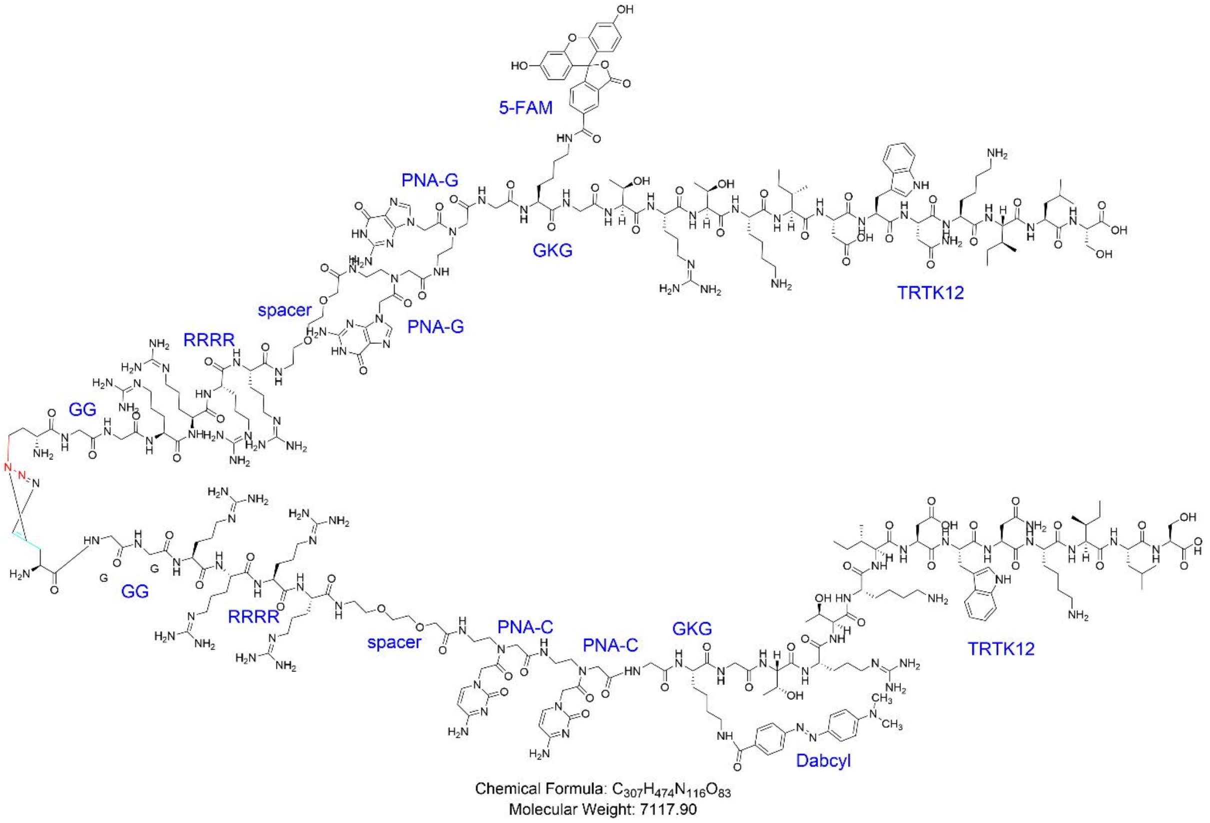

Figure S11. Chemical structure of the PNA-beacon bioreceptor, illustrating all involved moieties.
